# Molecular Hallmarks, Agronomic Performances and Seed Nutraceutical Properties to Exploit Neglected Genetic Resources of Common Beans Grown by Organic Farming in Two Contrasting Environments

**DOI:** 10.3389/fpls.2021.674985

**Published:** 2021-05-25

**Authors:** Pietro Sica, Francesco Scariolo, Aline Galvao, Domiziana Battaggia, Carlo Nicoletto, Carmelo Maucieri, Fabio Palumbo, Dorcas Franklin, Miguel Cabrera, Maurizio Borin, Paolo Sambo, Gianni Barcaccia

**Affiliations:** ^1^Department of Crop and Soil Sciences, University of Georgia, Athens, GA, United States; ^2^Department of Agronomy Food Natural Resources Animals and Environment (DAFNAE), University of Padova, Padua, Italy

**Keywords:** *Phaseolus vulgaris* L., local varieties, genetic diversity, yield, seed quality traits

## Abstract

Common bean (*Phaseolus vulgaris* L.) is an essential source of food proteins and an important component of sustainable agriculture systems around the world. Thus, conserving and exploiting the genetic materials of this crop species play an important role in achieving global food safety and security through the preservation of functional and serependic opportunities afforded by plant species diversity. Our research aimed to collect and perform agronomic, morpho-phenological, molecular-genetic, and nutraceutical characterizations of common bean accessions, including lowland and mountain Venetian niche landraces (ancient farmer populations) and Italian elite lineages (old breeder selections). Molecular characterization with SSR and SNP markers grouped these accessions into two well-separated clusters that were linked to the original Andean and Mesoamerican gene pools, which was consistent with the outputs of ancestral analysis. Genetic diversity in the two main clusters was not distributed equally the Andean gene pool was found to be much more uniform than the Mesoamerican pool. Additional subdivision resulted in subclusters, supporting the existence of six varietal groups. Accessions were selected according to preliminary investigations and historical records and cultivated in two contrasting Venetian environments: sea-level and mountain territories. We found that the environment significantly affected some nutraceutical properties of the seeds, mainly protein and starch contents. The antioxidant capacity was found significantly greater at sea level for climbing accessions and in the mountains for dwarf accessions. The seed yield at sea level was halved than mountain due to a seeds reduction in weight, volume, size and density. At sea level, bean landraces tended to have extended flowering periods and shorter fresh pod periods. The seed yield was positively correlated with the length of the period during which plants had fresh pods and negatively correlated with the length of the flowering period. Thus, the agronomic performance of these genetic resources showed their strong connection and adaptation to mountainous environments. On the whole, the genetic-molecular information put together for these univocal bean entries was combined with overall results from plant and seed analyses to select and transform the best accessions into commercial varieties (*i.e.*, pure lines) suitable for wider cultivation.

## Introduction

Legumes play an important role in addressing issues related to the environment, health, and food security and are also important due to their health benefits, such as preventing and helping manage hypercholesterolemia, hypertension (Arnoldi et al., 2015), obesity, diabetes, and coronary conditions (Calles, 2016). They are also a critical and affordable source of plant-based proteins, vitamins, and essential minerals such as calcium, magnesium, and zinc, contributing to the food security and nutrition of people around the world, especially subsistence smallholder farmers in developing countries (Calles, 2016). In developed countries, vegetarians, vegans, and individuals following flexitarian diets tend to increase, and legumes are recommended as the main plant-based protein source (Nelson et al., 2016).

The common bean (*Phaseolus vulgaris* L.) is a diploid (2n = 2x = 22) annual species belonging to the *Fabaceae* family grown worldwide for its edible green pods and dry seeds. Given the relative simplicity and the small dimension (650 Mb) of its genome, *P. vulgaris* provides a useful model for studying closely related species of agronomic interest. It is a predominantly self-pollinating plant, with occasional occurrence of insect-mediated cross-pollination (Rendón-Anaya et al., 2017). Breeding strategies for the common bean rely on the selection of homozygous individuals for the development of pure lines of high agronomic value.

The domestication process of the common bean was a unique process that occurred in two geographically distinct regions simultaneously and in two partially isolated gene pools: Mesoamerican and Andean. Genetic evidence suggests that Mesoamerica is the center of origin of the common bean, whereas the Andean population was derived as a consequence of a strong predomestication bottleneck. Despite having undergone independent domestication processes, both gene pools are partially sexually compatible and morphologically similar. Differences between the two gene pools have been revealed using different molecular markers, such as random amplified polymorphic DNA (RAPD) (Johns et al., 1997; Beebe et al., 2000), amplified fragment length polymorphisms (AFLP) (Tohme et al., 1996; Beebe et al., 2001; Pallottini et al., 2004), and microsatellites or simple sequence repeat (SSR) markers (Díaz and Blair, 2006). More recently, single-nucleotide polymorphism (SNP) markers have been used to characterize genotype and haplotype diversity in common bean accessions, assaying both nuclear (Ariani et al., 2016, 2018; Rendón-Anaya et al., 2017; Kuzay et al., 2020) and plastidial genomic regions (Nicolè et al., 2011).

Varieties of *P. vulgaris* are distributed worldwide and are cultivated in the tropics, subtropics, and temperate zones (Hidalgo, 1988), showing great variability in terms of agronomic performance, seed size, shape and color, the relative duration of the reproductive cycle, and many other qualitative and quantitative traits (Rodiño et al., 2003). This diversity enabled its cultivation in a wide range of cropping systems and environments, such as China, Eastern Africa, the Americas, the Middle East and Europe, with more than 40,000 varieties (Jones, 1999).

The first introduction of the common bean from Central/South America into Western Europe most likely took place in the sixteenth century (Zeven, 1997). A peculiarity of the European population of *P. vulgaris* is the high proportion (44%) of Mesoamerican and Andean hybrids. This could be explained by the presence of different landraces, which are traditionally cultivated in proximity to each other, facilitating occasional outcrossing and gene flow (Angioi et al., 2010). In Italy, beans made their first appearance in 1515 in a painting of Giovanni di Udine (Albala, 2007), whereas they were officially mentioned in historical documents by 1532, which was later recognized as the year of its introduction to the Italian Peninsula. In particular, in the Veneto region, the diffusion of the common bean occurred quickly, and currently, the cultivation of this pulse still has great economic relevance, especially in Belluno Province (Piergiovanni and Lioi, 2010). Bean cultivation gave rise to a long tradition that allowed the evolution of many landraces adapted to microclimates in restricted areas, representing a pastiche of cultures and traditions that provide an irremissible good for Italy that is being used in low-environmental impact agriculture (Venora et al., 2009). However, as for other Venetian crops (Palumbo et al., 2017a,b), local accessions have been gradually substituted by superior and genetically uniform commercial varieties (Spagnoletti Zeuli et al., 2004; Venora et al., 2009). In particular, after the 1950s, the large scale of breeding programs and the fast disappearance of landraces caused the disappearance of an unknown number of populations and the marginalization of others in private gardens. The commercial relevance of these landraces is generally limited, as the product is often sold only in local markets and appreciated and used in the preparation of local dishes (Piergiovanni et al., 2000). This fact and the possibility of using these rustic and vigorous genotypes in breeding projects increase the importance of collecting and preserving those local accessions in germplasm, *in-situ*, or *ex-situ*, contributing to the improvement of food crops and preserving their genetic diversity (Piergiovanni and Laghetti, 1999). Collecting, conserving, and preserving niche landraces from the Veneto region are some of the objectives of this study.

Although Veneto is not one of the primary domestication centers of the common bean, the collected material can be considered an example of serendipitous value from conserving landraces in a variety of places. Through their preservation, these niche landraces have undergone autochthony with minor adaptations, as they have been cultivated in isolation for centuries in a sort of ecotypization process. This process selected accessions, and peculiar gene combinations were able to achieve maximum adaptation to the new pedoclimatic and anthropic conditions. Long-term germplasm utilization and conservation practiced yearly by farmers, along with natural evolutionary processes, have therefore brought about the constitution of a regional multispecies germplasm that includes, among others, common beans (Supplementary Material).

High-quality and authentic agri-food products, which are mostly recognized as coming from organic farming or labeled with geographical indications, are increasing in Europe as consumers consider the quality and its association with agro-ecological characteristics as some of the most important purchasing factors. Italian agriculture for quality food production is widely spread across the national territory, highlighting a major role in the European context that is favored due to its great variety in terms of pedoclimatic and orographic conditions, together with its cultural and traditional approach to food (Dal Ferro and Borin, 2017). Organic farming systems cover approximately 15.2% of the national utilized agricultural area (Eurostat, 2019), generating almost 7.5% of the Italian agriculture value production (Istat, 2019). In this scenario, interest in preserving and enhancing local genotypes to identify their agronomic and qualitative characteristics is evident. This is done to find varieties able to satisfy the main needs of organic farming that can often be considered pillars, namely, the rusticity of the genotypes and their ability to adapt to different environmental conditions and marginal contexts with reduced pedoclimatic performances. Furthermore, bean cultivation is fully part of this contest, considering its potential role not only in terms of the nutritional value of the grain produced but also in relation to the benefits that the cultivation of a legume can bring in terms of soil fertility, especially for organic agriculture, as recently stated by Regulation (EU) 2018/848.

In this work, 26 bean accessions – 13 ancient local varieties (Venetian farmer populations) and 13 improved old lineages (Italian breeder selections) – were assessed in two different environments of the Veneto region: in sea-level lowlands and in mountainous lands. In agronomic terms, the phenological development and yield were assessed. The nutritional values of the seeds were characterized based on the total phenols, antioxidant activity, protein and starch contents, and amino acid composition. Seeds were also characterized based on their physical properties, including weight, volume, density, length, width, and thickness. Finally, accessions were genetically characterized through molecular markers for assessing the population structure, genotype composition, and relationships among accessions. Overall, this information will be of great help for the socioeconomic valorization of ancient local genetic resources that are well characterized molecularly and for the implementation of cultivation and agronomic practices in organic agricultural systems.

## Materials and Methods

### Plant Materials

Accessions assessed in this study were selected from the germplasm bank of the Department of Agronomy, Food, Natural Resources, Animal and Environment (DAFNAE) of the University of Padova in Legnaro, Italy. Among the species conserved in the DAFNAE germplasm, *P. vulgaris* was represented by 48 accessions (Supplementary Table 1), of which 26 were selected and cultivated in both locations, sea level and mountain. A list of the names, identification numbers, some characteristics and pictures of the seeds of these 26 accessions is shown in Table 1. Of these 26 accessions, 13 were lowland and mountain-climbing Venetian local varieties (hereafter defined as farmer populations), and 13 were Italian old lineages (breeder selections), among which were seven dwarf and six climbing (Italian elite varieties) beans. Due to differences in the cultivation process, accessions were divided between dwarf and climbing.

**TABLE 1:** Identification numbers, names, type, and growth habit of the 26 common bean accessions assessed in this study.

Id	Name	Type	Growth		Id	Name	Type	Growth	
1	Mangiatutto rampicante	Italian pre-commercial	Indeterminate (climbing)	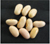	23	Gialet	Venetian landrace	Indeterminate (climbing)	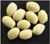
2	Borlotto nano A	Italian elite line	Determined (Dwarf)	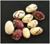	24	Posenati	Venetian landrace	Indeterminate (climbing)	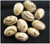
3	Borlotto nano B	Italian elite line	Determined (Dwarf)	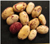	25	Semi-rampicante abruzzese	Venetian landrace	Indeterminate (climbing)	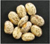
6	Fagiolo nano creso	Italian elite line	Determined (Dwarf)	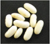	26	Fasol dela nonna	Venetian landrace	Indeterminate (climbing)	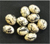
7	Blue lake sel. Gia	Italian pre-commercial	Indeterminate (climbing)	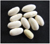	27	Maseleta rossa	Venetian landrace	Indeterminate (climbing)	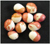
8	Anellino di Trento	Italian elite line	Determined (Dwarf)	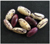	28	Zia Orsolina	Venetian landrace	Indeterminate (climbing)	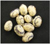
9	Anellino giallo	Italian pre-commercial	Indeterminate (climbing)	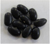	29	Meraviglia di Venezia	Venetian landrace	Indeterminate (climbing)	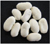
11	Bortollo lingua di fuoco 3	Italian pre-commercial	Indeterminate (climbing)	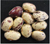	30	Secle	Venetian landrace	Indeterminate (climbing)	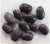
12	Blue lake a grano nero	Italian pre-commercial	Indeterminate (climbing)	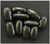	31	Della Clorinda	Venetian landrace	Indeterminate (climbing)	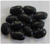
17	Fagiolo nano valdarno	Italian elite line	Determined (Dwarf)	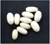	32	Pegaso	Venetian landrace	Indeterminate (climbing)	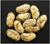
18	Coco nain blanc precoce	Italian elite line	Determined (Dwarf)	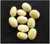	33	SC-iosela	Venetian landrace	Indeterminate (climbing)	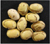
19	Tondino abruzzese	Italian pre-commercial	Indeterminate (climbing)	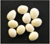	34	D’oro (val di fiemme)	Venetian landrace	Indeterminate (climbing)	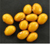
20	Verdone del piave	Italian elite line	Determined (Dwarf)	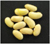	36	Maron	Venetian landrace	Indeterminate (climbing)	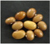

*Each is followed by a picture of its seeds on the right.*

### Experimental Sites and Environmental Conditions

Field trials were conducted between June and November 2019 in two different environments of the Veneto region—Legnaro (PD, Veneto Italy) at sea level (45°20′43.52″N, 11°57′11.56″E) and Asiago (VI, Veneto Italy) in the mountain (45°53′30.58″N, 11°33′43.58″E). In both locations, the planting layout used was 50-cm rows with 50 cm between rows, and approximately 150 cm between accessions. At sea level, 20 plants of each accession were transplanted in plots with dimensions of 5 m × 1 m. Climbing accessions, numbering 19 in total, were planted in five rows, each 44 m long. Seven dwarf accessions were transplanted in three rows. In the mountain, 10 plants of each accession were transplanted in plots of 2.5 m × 1 m. For the climbing accession, a structure to direct the plant vertical growth was constructed, consisting of four 2.1-m bamboo canes tied at the top, forming “hut” structures. Each plant was attached to one bamboo cane. Thus, in each parcel at sea level, 20 individuals were grouped into five groups of four plants, and in the mountains, the 10 plants were in two groups of four plants and in a pair of plants for three replications.

At sea level, the experiment was conducted at the Experimental Farm ‘L. Toniolo’ of the University of Padova at an altitude of 7 m above sea level. The soil at sea level is a eutric fluvisol (World Reference Base) and is composed of 65% silt, 15% sand, and 20% clay, with a pH of 8.15 (1:1 H_2_O). The organic matter was 1.77%, the total nitrogen was 0.11%, and the C/N ratio was 9.72. The total phosphorus determined by the Olsen method was 810 mg P_2_O_5_ kg^–1^, the potassium was 59.9 mg K_2_O kg^–1^, the magnesium was 247 mg kg^–1^, the calcium was 2,619 mg kg^–1^, the sodium was 26.1 mg kg^–1^ and the total sulfur was 408 mg kg^–1^. The cycle at sea level was from June 10th until October 25th (137 days), with an average temperature of 20.4°C. The maximum and minimum average temperatures were 28.4°C on 37.6°C on the 26th day and 7.7°C on the 135th day (Supplementary Material), and the cumulative rainfall was 236 mm.

On the mountain, the trial was conducted on a local farm at an altitude of 994 m. The soil on the mountain is a eutric cambisol (World Reference Base) and was composed of 45% silt, 15% sand, and 40% clay, with a pH of 6.77. The organic matter was 2.6%, the total nitrogen was 0.10%, and the C/N ratio was 15.11. The total phosphorus was 750 mg P_2_O_5_ kg^–1^, the potassium was 67.3 mg K_2_O kg^–1^, the magnesium was 201 mg kg^–1^, the calcium was 1,932 mg kg^–1^, the sodium was 18.1 mg kg^–1^ and the total sulfur was 550 mg kg^–1^. The cycle on the mountain was from June 24th until November 7th (136 days), with an average temperature of 14.1°C. The maximum and minimum average temperatures were 28.4°C on the 34th day and −1.4°C on the 117th day, respectively, and the cumulative rainfall was 512 mm. Maximum and minimum temperature trends during the experimental period followed the historical average trends at both sites (Supplementary Figure 1). In both locations, an irrigation system was installed, and water was supplied according to the plant needs.

### Cultivation Operations

At both locations, the experiments were carried out in soils that were not previously treated with chemical inputs (fertilizers, pesticides) for at least 5 years, thus enabling us to consider their conditions adequate for organic farming according to EU regulations. Cultivation operations were carried out primarily in the same way in both locations without any use of inputs not allowed for organic farming. The soil was prepared by harrowing (milling in a mountain plot), and subsequently, the organic fertilizer Biorex (pelleted manure) of Italpollina (derived from poultry litter) was distributed at a rate of 0.8 Mg ha^–1^. The fertilizer had the following composition: N 2.8%, P_2_O_5_ 2.5%, K_2_O 3.0%, organic carbon of biological origin 38%, organic matter 65%, C/N 13, and water 16%, with a pH of 7.

Seeds were sown in seedling starter trays (40 holes), arranging one seed in each hole and using fine peat-based Geo Substrate (PBE Substrates) containing 2–6 mm perlite, with a pH of 5–6 and an EC of 0.4 mS cm^–1^. Sowing was carried out on May 30 for the material destined for sea level and on June 10 for that to be used on the mountain. Although traditional agronomic practice includes direct sowing of beans, it was decided to sow the beans on the substrate and then proceed with transplanting due to the scarce availability of seeds of some accessions. This was done to ensure the emergence of enough seedlings to be transplanted in each location, avoiding the possible drawbacks caused by direct sowing in the field, i.e., the formation of a surface crust and attack by pathogens and parasites during germination.

After sowing, seedling starter trays were kept in a protected environment and watered regularly to ensure optimal substrate water content. Approximately 10 days after sowing, the seedlings had already developed the first two true leaves and had adequately colonized the media with the root system. Transplants were carried out during the first meteorologically useful window of the season (Supplementary Figure 1): on June 10 at sea level and on June 21 on the mountain.

The harvesting of pods was carried out gradually when they appeared completely dry on the plant. At sea level, this phase started in the second half of August and lasted until the end of October, while on the mountain, it started in the second half of September and ended in early November. The maturation of the pods was therefore scaled in a more accentuated way for climbing accessions that tend to have indeterminate growth.

### Genetic Characterization

In total, 193 genomic DNA samples were extracted from young leaves of 26 *P. vulgaris* using the DNeasy 96 Plant kit (Qiagen, Hilden, Germany) following the instructions provided by the supplier. After extraction, the DNA quality and quantity were evaluated using a NanoDrop 2000c UV-Vis spectrophotometer (Thermo Fisher, Pittsburgh, PA, United States). The DNA sample integrity was also checked by electrophoresis on a 2% agarose/1× TAE gel containing 1× Sybr^®^ Safe DNA gel stain (Life Technology, Carlsbad, CA, United States).

An initial number of 24 SSR markers was chosen from the literature (Yu et al., 2000; Blair et al., 2009; Angioi et al., 2010) based on their polymorphism information content (PIC), linkage map position, and sequence length. Tests on the amplification efficiency of the designed primer pairs were conducted in singleplex reactions on a subset of 8 samples of as many varieties. Amplifications were accomplished following the M13-tailed SSR method described by Schuelke (2000) and modified as reported by Palumbo et al. (2017a; 2017b) using 6-FAM, VIC, NED, and PET fluorophores. The 10 best SSR marker loci were selected and organized into two multiplexes based on the primer annealing temperature, amplicon size, amplification efficiency, and dimer formation tendency (Table 2). PCR was performed in a final volume of 20 μL containing 1x Platinum Multiplex PCR Master Mix (Thermo Scientific, Carlsbad, CA, United States), 5% GC Enhancer (Thermo Scientific), 0.25 μM of each tailed primer, 0.75 μM of each non-tailed primer, 0.5 μM of each labeled primer (Applied Biosystem, Carlsbad, CA, United States), 10 ng of DNA and sterile water. The fluorescently labeled PCR products were electrophoresed on an ABI 3730 DNA Analyzer (Applied Biosystems). Finally, the size of each fragment was determined by Peak Scanner software 1.0 (Applied Biosystems).

**TABLE 2:** List and basic information of the primer pairs for the Microsatellite (neutral) marker loci selected for DNA genotyping.

Marker	LG	Primer forward (5′-3′)	Primer reverse (5′-3′)	Motif	Size (bp)	PIC	Ta (°C)
BM200	1	TGGTGGTTGTTATGGGAGAAG	ATTTGTCTCTGTCTATTCCTTCCAC	(AG)10	221	0.89	56.0
AY1	1	ATCAGGGTCTGTCATGATCTG	CCTCCTCTCTTCTTGTTCCT	(AT)5	203	0.70	55.0
GATS91	2	GAGTGCGGAAGCGAGTAG	TCCGTGTTCCTCTGTCTGT	(GA)17	229	0.91	57.0
BM197	3	TGGACTGGTCGATACGAAG	CCCAGAAGATTGAGAACACCA	(GT)8	201	0.56	56.0
IAC52	4	TGCATGTATGTAGGCGGTTTA	GTGGCTTTTGCTTTTGTAGTCA	(GA)11	203	0.64	55.0
IAC66	4	AATCACATCTTTAACCCAACAGGT	TTCCACTCCCTCCCTATCT	(GA)10	282	0.86	56.0
BM183	7	CTCAAATCTATTCACTGGTCAGC	TCTTACAGCCTTGCAGACATC	(TC)14	149	0.84	55.5
BM210	7	ACCACTGCAATCCTCATCTTTG	CCCTCATCCTCCATTCTTATCG	(CT)15	166	0.88	56.4
PvM04	8	GGTTCCTCCTCCTTCTGC	GCGCCGTCTTTTTGGTAG	(TTC)10	210	0.77	56.0
PvAG001	11	CAATCCTCTCTCTCTCATTTCCAATC	GACCTTGAAGTCGGTGTCG	(GA)12	157	0.74	56.5

*Marker name, linkage group (LG), SSR motif, forward and reverse primer sequences, expected amplification fragment size (bp), Polymorphism Index Content (PIC), and temperature of annealing (Ta °C) are reported.*

The SSR raw data were analyzed with the POPGENE 32 software package v. 1.32 (Yeh et al., 1997), and the following statistics were calculated: the observed homozygosity (Obs_Ho), the average number of alleles per locus (na), the effective number of observed alleles per locus (ne) and the SSR allele frequencies. All statistics were calculated for both the SSR loci used and the 48 accessions analyzed. To determine the allele variability of the assessed marker loci, Nei’s index was calculated and assumed to be the polymorphism index content (PIC) (Palumbo and Barcaccia, 2018). Otherwise, considering the subpopulation later identified, the same index was used to express their heterozygosis grade.

Genetic similarity (GS) estimates were also calculated between individuals in all possible pairwise comparisons by applying Rohlf’s simple matching (SM) coefficient using NTSYS v2.1 software (Rohlf, 2000). The resulting similarity matrix was later used for the construction of a UPGMA dendrogram. The average similarity was also calculated within and among both the niche landraces and elite lineages, and a principal coordinates analysis (PCoA) graph was developed from the similarity matrix. Samples were then labeled on the basis of the results obtained by both STRUCTURE software, which was used for ancestry group reconstruction, and the UPGMA dendrogram.

From the initial core collection including 193 samples, a panel of 41 accessions of *P. vulgaris* belonging to 25 varieties (variety 19 was not considered due to the high number of missing loci) was selected for haplotyping analysis based on SNP variants. Samples were chosen to be representative of the internal variability of each population and with 100% homozygosity. This is preferred to obtain single haplotypes and because pure lines are used in breeding programs for the establishment of patented and stable varieties.

The nuclear target genes to be used for DNA haplotyping were preliminarily obtained from the scientific literature (Schlötterer and Pemberton, 1994; Rakoczy-Trojanowska et al., 2004; Kumar et al., 2012), choosing single-copy loci with functions related to plant and seed development and associated with abiotic stress resistance or tolerance. In particular, the final set of target genes was selected from among those reported by selected papers (McConnell et al., 2010; Nanni et al., 2011; Goretti et al., 2014). For each of these target genes, the pair of primers was designed and optimized to obtain fragments suitable for the subsequent sequencing analysis. The investigated marker regions are coding and/or noncoding fragments of eleven genes: β-1,3-endoglucanase (β-EG), heat shock transcription factor (HSF), β-glucan binding protein (β-GBP), phosphoenolpyruvate carboxylase (PEPC), late embryogenesis abundant protein (LEA), serine/threonine kinase (STK), nitrate reductase (NR), linoleate lipoxygenase (LOX), beta-amylase (β-AM) (Goretti et al., 2014), histone H4 (H4) (McConnell et al., 2010), and shatterproof (SHP) (Nanni et al., 2011). Whenever the indicated amplified fragment was larger than 900 bp, a new primer pair was designed (in bold in Table 3) to obtain a fragment shorter than 900 bp in substitution of the original fragment. Using the *P. vulgaris* reference genome hosted in the Phytozome database^1^, primers were designed to match the exons of the selected genes, and where possible, introns were included to maximize the genetic variation estimate. Primer-BLAST^2^ was used for primer design, and primers were purchased from Invitrogen (Invitrogen, Carlsbad, CA, United States).

**TABLE 3:** List and basic information on the Mendelian loci (expressed regions) selected for DNA haplotyping, including genomic localization (linkage group, LG), gene name, sequences of primer pairs used for PCR amplification, size of the amplified fragment (bp), temperature of annealing (Ta °C), and primer used for sequencing (highlighted in bold) in order to obtain amplicons <900 bp in length in substitution to the original ones.

LG	Gene	Primer forward	Primer reverse	Size	T_*c*_	Sequencing
LG01	Beta-1,3-endoglucanase^1^	CAAACAAATGGGTGCAAGACAA	TCATGCTCTGGATGCTTCTG	670	59°	FW
LG02	Heat shock transcription factor^1^	CTTGTTTGGGTATTTGGGGTTA	AACCGGCTTCCGTCTATG	794	57°	FW
LG03	Histone H4^2^	ATCAGCCATGTCTGGAAGAGGAAA	TGCTTGAAAATGTCCAAATCATTC	502	57°	RV
LG04	Beta-glucan binding protein^1^	**TGAGCCTTGTACTTCCTACCC**	**AAGTTTAGTTCTTGTTACCCCGTG**	683	57°	FW
LG05	Phosphoenolpyruvate carboxylase^1^	GCTGCAAGAGATGTACAACCAA	ATAACGAAAGGGAAGATGGGTGA	618	57°	FW
LG06	Shatterproof^3^	**TTTGCTGATGTCGAGTTCATGC**	**ATTGTTGTTGTTGTTGTTGAGCTC**	531	57°	FW
LG07	Late embryogenesis abundant protein^1^	**GAGATGAAGGATGCGGCGAA**	**TCCTCCAGTTTCCTCCTTGC**	753	63°	FW
LG08	Serine/Threonine kinase^1^	**TCCTGAAACTCAGCCCCAAG**	**CCTGAGATCCGTCAACACCC**	640	57°	RV
LG09	Nitrate reductase^1^	**TGTGGAGCGTCTGGAGAAAC**	**TGCACACGTTCGTCTTCACT**	889	57°	FW
LG10	Linoleate 13S-lipoxygenase^1^	**TTAGCCCCATACCAGTGCTT**	**TGAACAATCTGTTACCATGCAGT**	628	59°	RV
LG11	Beta-amylase^1^	TGGTCCACTTTTGGCATCT	CCACAAAATCAAGGATGGGAAT	685	57°	RV

*^1^Goretti et al. (2014); ^2^McConnell et al. (2010); ^3^Nanni et al. (2011).*

The PCR mixes were composed of 10 μl Mango mix (Bioline, 2020), 0.25 μM of both primers, 20 ng genomic DNA, and sterile distilled H_2_O to reach a final volume of 20 μl per reaction. Amplifications were carried out using the following conditions: initial denaturation for 2 min at 95°C, then 40 cycles of 30 s at 95°C, 30 s at the annealing temperature, and 1 min at 72°C, followed by one last final extension for 10 min at 72°C. Annealing temperatures specific to each primer pair can be found in Table 2. Amplification products were purified using exonuclease I and FastAP thermosensitive alkaline phosphatase (Thermo Fisher) according to the manufacturer’s protocol for PCR product clean-up before sequencing. After purification, gene fragments were analyzed using Sanger sequencing by performing a single-strand reaction on ABI 3730XL with PHRED20.

Sequencing data were analyzed using Geneious 3.6.1 (Geneious, 2020), and the resulting sequences were aligned using the Muscle algorithm implemented in MEGA-X (Kumar et al., 2018). The detected SNPs were then classified as synonymous or nonsynonymous mutations. Haplotype reconstruction was performed by identifying all the unique combinations of genes existing in the core collection. In addition, the haplotype number (Hn) and haplotype diversity (Hd) were estimated according to Nei (1987).

Polymorphic loci were analyzed using NTSYS v2.1 (Rohlf, 2000) software to obtain the genetic similarity (GS) estimate in a pairwise comparison using the simple matching coefficient. The resulting similarity matrix was used for the development of a UPGMA dendrogram using hierarchical clustering. To reconduct the two main clusters highlighted by the UPGMA tree within the Andean or Mesoamerican gene pool, sequences of accessions with known geographical origins were retrieved from studies by Nanni et al. (2011) and Goretti et al. (2014). Information was not available for all the analyzed markers and was available only for β-EG, β-GBP, and SHP. The genetic sequences were aligned with MEGA-X using the Muscle algorithm, and the UPGMA tree was then generated using 1000 bootstrap and Kimura 2-parameter models.

Population structure analysis of the core collection was performed using STRUCTURE software, which exploits a systematic Bayesian clustering approach by applying Markov chain Monte Carlo (MCMC) estimation (Pritchard et al., 2000), which compares the molecular marker data belonging to each accession among themselves to infer their membership in a series of putative clusters. The simulation was performed assuming the admixture model, with no *a priori* population information. The SNP data were analyzed with 106 iterations and a burning period of 2⋅105, and ten replicate runs were executed with the value of K ranging between 1 and 16. The most likely K value was estimated using ΔK (Evanno et al., 2005) and was considered for the assignment to an ancestral group.

### Agronomic and Morphological Characterizations

#### Phenological Assessment

At sea level, for each accession, ten plants were identified from among those positioned more internally in the parcel (considering the pairs, five in one row and five in the other) and kept as samples for phenological observations until collection. On the mountain, with only ten plants per accession, six plants were used as models for each parcel, following the same criterion adopted at sea level. Phenological surveys were carried out weekly at sea level and every 2 weeks on the mountain.

The BBCH scale based on Zadoks et al. (1974) and extended by Meier (2018) was used to discriminate the phenological stages. This implies a decimal scale for the description of phenological development. In this study, only 8 of the 10 principal growth stages were considered: (0) germination (0–09); (1) leaf development (10–19); (2) formation of side shoots (21–29); (5) inflorescence emergence (51–59); (6) flowering (60–69); (7) development of fruit (71–79); (8) ripening of fruit and seeds (81–89); and (9) senescence (97–99). For practical reasons, the BBCH stages were then merged as follows: vegetative phase (0–29), flowering phase (51–69), fresh pod period (71–79), and dry pod period (81–99). The identification codes obtained from the field observations were subsequently averaged to obtain a single value referring to the individual parcel.

#### Growing Degree Days

The number of cumulative GDD (growing degree days), which is also expressed as the thermal sum (ΣGDD), was calculated by summing the positive values of Eq. 1 for each day and considering a base temperature of 10°C (Jenni et al., 2000).

(1) $$GDDx=\left(\frac{Tmax+Tmin}{2}\right)-Tbase$$

Where GDD*x* is the growing degree day on day *x*, Tmax and Tmin are the maximum and minimum average air temperature of day *x*, respectively, and Tbase is the base temperature. The temperature at both locations was monitored by weather stations from the Regional Agency for Environmental Prevention and Protection of Veneto (ARPAV).

#### Seed Production

After maturing and drying in the plant, pods were harvested and completely dried at room temperature (until reaching approximately 10% moisture). The production was expressed in grams of dry grain per plant.

#### Seed Physical Properties

Seeds of each accession and location were assessed for the seed weight, bulk density, volume, and imbibition capacity, according to Bishnoi and Khetarpaul (1993) and Shimelis and Rakshit (2005). For each accession, three replicates of 100 seeds from the mountain and sea level were collected and weighed, giving a weight of 100 seeds in grams. Of these, ten were collected and inserted into a 100-mL graduated cylinder containing 50 mL of distilled water. After the seeds were added to the cylinder, the variation in the volume was considered the volume of 10 seeds. Density was expressed in g mL^–1^.

### Seed Nutraceutical Characterization

#### Total Phenols and Antioxidant Capacity

Freeze-dried bean samples (0.2 g) were homogenized in methanol (20 mL) with an Ultra Turrax T25 until reaching a uniform consistency at 13.500 rpm. The samples were filtered (filter paper, 589 Schleicher), and appropriate aliquots of the extracts were assayed by a Folin Ciocalteu (FC) assay for the total phenol (TP) content and by a ferric reducing antioxidant power (FRAP) assay for the total antioxidant activity.

The content of TP was determined using the FC assay with gallic acid as the calibration standard by a Shimadzu UV-1800 spectrophotometer (Columbia, MD, United States). The FC assay was carried out by pipetting 200 μL of bean extract into a 10-mL polypropylene tube. This was followed by the addition of 1 mL of FC reagent. The mixture was vortexed for 30 s, and 800 μL of filtered 20% sodium carbonate solution was added after 1 min and before 8 min from the addition of the FC reagent. This was recorded as time zero. The mixture was then vortexed for 30 s after the addition of sodium carbonate. After 2 h at room temperature, the absorbance of the colored reaction product was measured at 765 nm. The content of TP in the extracts was calculated from a standard calibration curve built with different concentrations of gallic acid, ranging from 0 to 600 μg mL^–1^ (correlation coefficient: R^2^: 0.9982). The results were expressed on the basis of mg of gallic acid equivalents per kg (mg GAE kg^–1^) of dry bean powder (Singleton et al., 1999).

Ferric reducing antioxidant power reagent was prepared fresh so that it contained 1 mM 2,4,6 tripyridyl-2-triazine and 2 mM ferric chloride in 0.25 M sodium acetate at pH 3.6 (Benzie and Strain, 1996). A 100-μL aliquot of the methanol extract prepared as described above was added to 1900 μL of FRAP reagent and accurately mixed. After leaving the mixture at 20°C for 4 min, the absorbance at 593 nm was determined. Calibration was performed against a standard curve (0–1200 μg mL^–1^ ferrous ion) produced by the addition of freshly prepared ammonium ferrous sulfate. FRAP values were calculated as mg mL^–1^ ferrous ion (ferric reducing power) from three determinations and are presented as mg of Fe^2+^ (ferrous ion equivalent) kg^–1^ dw.

#### Amino Acids Composition

Determination of the amino acid content was carried out in accordance with the provisions of the European Pharmacopoeia 5.0 – 2.2.56. Amino acid analysis – Protein hydrolysis – Method 1 for hydrolysis and from the European Pharmacopoeia 5.0 – 2.2.56. Amino acid analysis – Methodologies of amino acid analysis: general principles – Method 5 and Method 7 for derivatization. HPLC identification and quantification were performed following Agilent ZORBAX Eclipse AAA – Technical Note (publication number 5980-1193).

#### Starch Content

The starch content was determined using the OAC Official Method 996.11 Starch (total) in cereal products and the AOAC Official Method 979.10 Starch in Cereals. Following the University of Florida, IFAS, Bulletin 339-2000 “Starch Gelatinization and Hydrolysis Method” Boehringer Mannheim, Starch determination, cat. N ° 207748 was the method adapted for chromatographic analysis.

#### Protein Content

Crude protein was determined by the analysis of the nitrogen content according to the semi-micro Kjeldhal technique (Kjeltec-System Foss Tecator) (Hjalmarsson and Akesson, 1983). The protein content was calculated by multiplying the N content by a factor of 6.25 (Adler-Nissen, 1986).

### Statistics

Data obtained from the various findings were separated between climbing and dwarf accessions due to differences in growth habits and production systems. To assess the effect of the cultivation environment, mountain and sea level (E), accession (A), and their interaction (E × A), analysis of variance was performed independently for each typology, climbing and dwarf. The differences between means were assessed by Tukey’s HSD test for *p* < 0.05. A principal component analysis (PCA) and a multivariate analysis performed to determine the correlations between the variables assessed in this study. All previously cited statistical analyses were performed in the software JMP Pro 15 (SAS – JMP Kuala Lumpru Malaysia). Excel and SigmaPlot 11.0 were used to prepare the graphs.

## Results

### Genetic Characterization

#### SSR Marker-Based Genotyping

The first part of this study aimed to identify the most suitable SSR markers for the characterization of the common bean core collection conserved in the DAFNAE germplasm bank. Ten of the 24 initially selected markers were chosen for further analyses since preliminary tests exhibited easy scorability, a marked attitude to be amplified in multiplex PCRs and the highest polymorphism information content (PIC) coefficients.

Descriptive statistics of genetic diversity calculated for the 10 marker loci exploited for the analyses are reported in Table 4. The mean number of observed alleles per locus (na) was 5.8, with values ranging from 2 (AY1) to 11 (GATS91). The effective number of alleles (ne) for the analyzed accessions ranged between 0.52 (AY1) and 6.69 (GATS91), with a mean value equal to 3.55. Each microsatellite locus scored high levels of observed homozygosity, ranging from 0.98 (IAC52 and IAC66) to 1.00, with an average observed homozygosity of 0.99. The PIC coefficients were also calculated using the marker allele frequencies at each locus to determine the discriminant ability of each marker locus among the different genotypes. With a minimum value of 0.56 (BM197) and a maximum value of 0.91 (BM183), the panel of selected microsatellite markers proved to be hypervariable and highly informative.

**TABLE 4:** Descriptive statistics of genetic diversity calculated for each of the 10 SSR marker loci analyzed in the common bean core collection.

Locus	na	ne	obs_Ho	PIC
IAC52	6	3.84	0.98	0.89
AY1	2	1.50	0.99	0.70
BM183	4	1.77	0.99	0.91
BM197	3	1.68	0.99	0.56
IAC66	7	2.26	0.98	0.64
BM210	6	4.89	1.00	0.86
PvM04	8	4.83	1.00	0.84
BM200	7	4.50	0.99	0.88
Pvag001	4	3.52	1.00	0.77
GATS91	11	6.69	1.00	0.74
Mean	5.8	3.55	0.99	0.78

*The number of observed alleles (na), the number of effective alleles (ne), observed homozygosity (obs_Ho), and the polymorphic information content (PIC) are reported for each SSR marker locus investigated.*

Supplementary Figure 2 shows the mean genetic similarity matrix calculated within and among the 25 populations. Values on the diagonal representing the genetic similarity of each population showed an overall very high uniformity – 15 populations scored 100% identity, and only two populations showed a genetic similarity below 0.95 (accessions “Blue lake a grano nero” and “Pegaso”).

**FIGURE 1:**
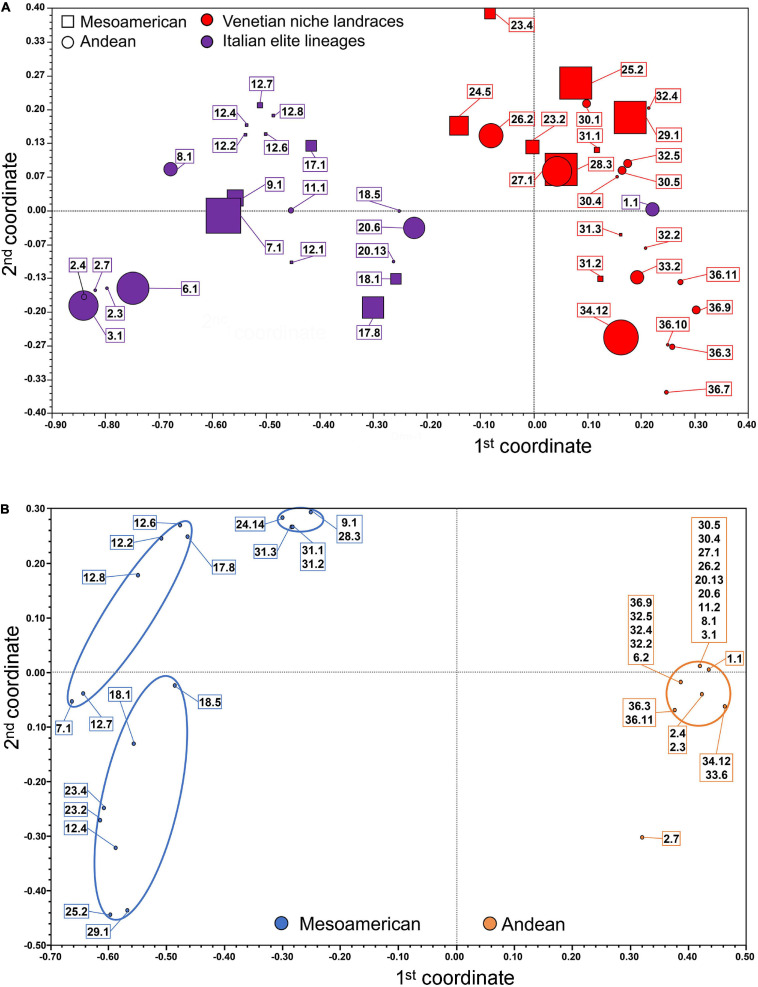
Principal Coordinate Analysis of the common bean accessions based on SSR and SNP markers: **(A)** Bidimensional centroids of the total 193 analyzed samples (symbol size is proportional to the number of samples scoring full genetic identity). **(B)** Bidimensional clusters of the 41 common bean unique genotype accessions identified through SSR markers and analyzed using SNP markers for the eleven chosen genes.

Genetic relationships among common bean accessions were further studied using principal coordinate analysis (Figure 1A). Individual unique genotypes were differently classified and discriminated, depending on whether they were assigned through the subsequent haplotyping analysis to the Andean or Mesoamerican gene pools and to Venetian farmer’s varieties or Italian breeder’s lineages (please note that symbol dimensions are proportional to the number of individuals sharing 100% similarity and hence are characterized by the same coordinates). The vast majority of populations represented by two or more subgroups are located in proximity to each other, forming almost isolated subgroups (for example, accession 12 “Blue lake a grano nero” and 36 “Maron”). Considering the first discriminant coordinate, there is a clear separation between the elite lineages and the niche landraces (with the only exception of genotype 1.1 from population “Mangiatutto rampicante”). Taking into consideration the second discriminant coordinate, the distinction is less clear (Figure 1A). It is, however, possible to highlight a trend in which the genotypes with Mesoamerican origin, as subsequently determined from the SNP variant analysis, possess a generally higher coordinate value for the second dimension compared to the genotypes of Andean origin, even with a large and shared area of centroids (Figure 1A). A total of 41 genotypically different individuals was then chosen as representative of the genetic diversity of the entire core collection and used in the subsequent haplotyping analyses, as they showed 100% homozygosity and similarities to each other lower than 100%.

#### SNP Variant-Derived Haplotyping

The subset of 41 unique genotypes belonging to 25 of the chosen 26 populations (accession 19 exhibited a significant amount of missing data, so its haplotype characterization was not reported in this study) was analyzed for 11 target genes using Sanger sequencing to investigate and characterize their haplotypes. Each gene is a single copy located on a distinct linkage group and is related to traits of agronomic interest or linked to loci known to be under selective pressure during the domestication process. The primer pairs that were used in this study revealed good PCR efficiency, and were able to work with a 100% success rate in *Phaseolus* genomic DNA samples and resulted in the amplification of single genomic regions (Supplementary Figure 3).

Sequenced fragments ranged between 409 and 818 bp, for a cumulative length of 6,533 bp composed of 55.5% exonic and 44.5% intronic regions. Based on sequence analysis, all the gene regions revealed 100% homozygosity, which was in agreement with the results obtained from microsatellite marker analysis. By aligning the DNA sequences of these target genes, in total, 48 SNP variants were detected, as well as 8 INDELs, which were distributed unequally across the DNA fragments. All the polymorphic sites found were biallelic, with the exception of the third single point variant of the LOX gene, where an adenine was either substituted with a thymine or deleted. The marker with the highest variability was the intronic region of the shatterproof (SHP) gene, with 10 SNP variants and 3 INDELs (1, 4, and 7 nucleotides long, respectively), whereas the β-AM gene was monomorphic in the investigated core collection. The occurrence of polymorphisms varied significantly based on the region taken into consideration – in exons, 0.47% of the nucleotides were polymorphic, while in introns, the occurrence of variants was three times higher, equaling 1.55%. This discrepancy was expected since noncoding regions are known to be neutral and thus retain point mutations, and they usually show higher diversity. Considering only the SNP variants detected in exon regions, it is worth mentioning that 8 out of 17 proved to be nonsynonymous mutations. Additional information on the SNP distribution and classification can be found in Supplementary Table 2.

#### Genetic Similarity and Genetic Structure Analyses

**FIGURE 2:**
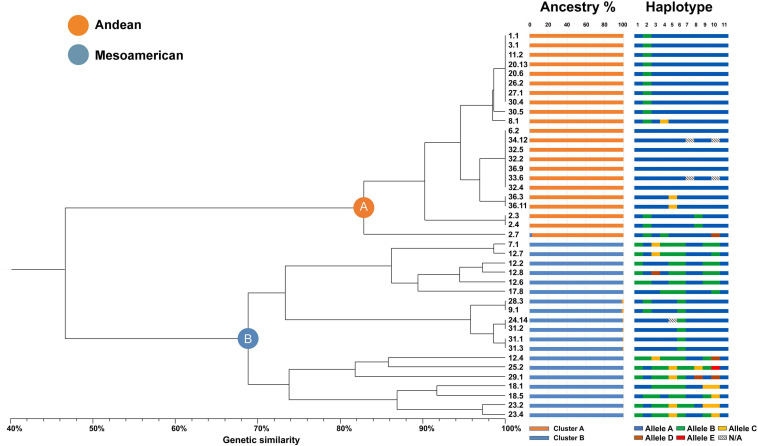
UPGMA dendrogram with all the common bean accessions analyzed in this study grouped in two main clusters **(A,B)** and several subclusters. The ancestry colored bars refer to the percentage of ancestral membership estimated by STRUCTURE analysis (for *k* = 2). The haplotype colored bars refer to the allele combinations found for each of the samples: individual bars are split into eleven blocks as the number of sequenced genes and each bar as a whole represents a multi-locus haplotype including 11 specific alleles (dashed blocks indicate missing data).

Molecular data of the polymorphic loci were used to calculate the genetic similarity matrix with the simple matching coefficient for all pairwise comparisons between the 41 individual genotypes. A UPGMA dendrogram, as shown in Figure 2, was then generated to investigate the relationships among genotypes and varieties.

All the accessions were grouped into two distinct and well-separated branches labeled A and B, respectively. Each of these two groups scored high mean genetic similarity, especially in cluster A, wherein all 22 genotypes were divided into seven subgroups. Moreover, 16 subgroups were found in cluster B, wherein the 19 genotypes showed lower mean genetic similarity. Notably, genotypes belonging to the same population were consistently assigned to only one of the two main clusters, even if placed in different minor branches. In total, 14 varieties were assigned to cluster A, and 11 varieties were associated with cluster B.

The least homogeneous population, accession 12 “Blue lake a grano nero,” was represented by five genotypes and was assigned to cluster B, contributing to the higher variability in this group. It is worth mentioning that in three cases, in both clusters A and B, individuals were present belonging to both Venetian niche landraces and national lineages grouped together with full haplotype genetic identity (e.g., samples 1.1 and 20.13 in cluster A and samples 9.1 and 28.3 in cluster B). The ancestral membership of the core collection was investigated using STRUCTURE software by the estimation of ΔK. This result suggested that the core collection most likely originated from two genetically distinct ancestors (K = 2 as the most likely value), as shown in Figure 2, where each genotype is represented by a histogram divided into two segments that are proportional to the membership to ancestor 1 or 2. This resulted in a clear division into two distinct and highly uniform groups composed of 22 and 19 accessions each, which was in agreement with the current evolutionary model of modern common beans derived from two distinct gene pools, Andean and Mesoamerican.

Then, we demonstrated that the two main clusters highlighted by the UPGMA tree analysis and supported by the STRUCTURE ancestry analysis do correspond to Andean or Mesoamerican origins. To interpret the results, the genetic sequences were aligned with accessions of known geographical origin and gene pool identity among those available on the NCBI database for the analyzed loci. Samples assigned to cluster A univocally belonged to the Andean gene pool, similar to those in cluster B to the Mesoamerican pool, supporting the hypothesis of the two gene pools of origin.

For 10 of the 11 regions sequenced, numbers of alleles (meaning specific combinations of polymorphic positions) ranging from 2 to 5 were identified (Table 3). Since recombination is unlikely to occur in closely linked loci, SNP variants are instead inherited together as a single unit, and the unique combinations found were limited: 2 alleles for β-EG, HSF, SHP, and LEA; 3 alleles for β-GBP, PEPC, and NR; 4 alleles for H4; and 5 alleles for STK and LOX. β-AM was monoallelic. In H4, β-GBP, LEA, STK, NR, and LOX SNP combinations were uncommon among the examined genotypes, with an incidence below 5%. The relative values are reported in Table 5.

**TABLE 5:** Relative frequency (%) of the SNP-derived allele variants found in the common bean core collection for each of the 11 target genes.

Genes	β-EG	HSF	H4	β-GBP	PEPC	SHP	LEA	STK	NR	LOX	β-AM
Allele A	75.60	53.70	75.60	73.20	61.00	53.70	90.20	90.30	75.60	61.00	100.00
Allele B	24.40	41.50	14.60	24.40	22.00	46.30	4.90	4.90	19.50	14.60	
Allele C			7.30	2.40	14.60			2.40	4.90	9.80	
Allele D			2.40					2.40		7.30	
Allele E										2.40	
S	3	3	7	3	3	22	1	8	2	19	0
Hp n	2	2	4	3	3	2	2	4	3	5	1
Hp d	0.68	0.77	0.85	0.80	0.85	0.75	0.59	0.80	0.80	0.92	0.00

*The number of polymorphic sites at each locus (S), the relative number of identified alleles (Hp n), and Nei’s haplotype distance (Hp d) (Nei, 1987) are reported.*

The various SNP combinations were arranged in 21 different multilocus haplotypes, as reported in Table 6. In agreement with the previous analysis, cluster A was characterized by higher genetic uniformity, and most of the accessions were represented by a single haplotype (Haplo_01), while cluster B showed wider genetic variability with 14 different haplotypes (Haplo_08-21). Particularly relevant in terms of protein functionality are the 8 SNP variants identified as responsible for amino acid substitutions (please note that all missense mutations are marked with an asterisk in the consensus sequence, see Table 6).

**TABLE 6:** Relative haplotype number (Hp n) and haplotype distance (Hp d) (Nei, 1987) of the SNP-derived allele variants found in the core collection for each of the 11 target genes and for each of the four identified clusters, based on accession identity (farmer populations and breeder selections) or ancestry identity (Andean and Mesoamerican), and in total.

Resources/Genes		β-EG	HSF	H4	β-GBP	PEPC	SHP	LEA	STK	NR	LOX	β-AM
Venetian niche populations	Hp n	2	2	2	2	2	2	2	3	3	4	1
	Hp d	0.65	0.68	0.65	0.65	0.75	0.74	0.67	0.73	0.75	0.87	0.00
Italian elite lineages	Hp n	2	2	4	3	2	2	1	2	3	4	1
	Hp d	0.71	0.73	0.87	0.83	0.75	0.75	0.00	0.59	0.83	0.91	0.00
Andean lineages	Hp n	1	2	1	3	2	1	1	2	1	2	1
	Hp d	0.00	0.74	0.00	0.72	0.58	0.00	0.17	0.58	0.00	0.63	0.00
Mesoamerican lineages	Hp n	2	2	4	2	3	1	2	3	3	5	1
	Hp d	0.75	0.69	0.92	0.75	0.89	0.00	0.59	0.73	0.86	0.95	0.00
Total	Hp n	2	2	4	3	3	2	2	4	3	5	1
	Hp d	0.68	0.77	0.85	0.80	0.85	0.75	0.59	0.80	0.80	0.92	0.00

### Phenological Assessment

**FIGURE 3:**
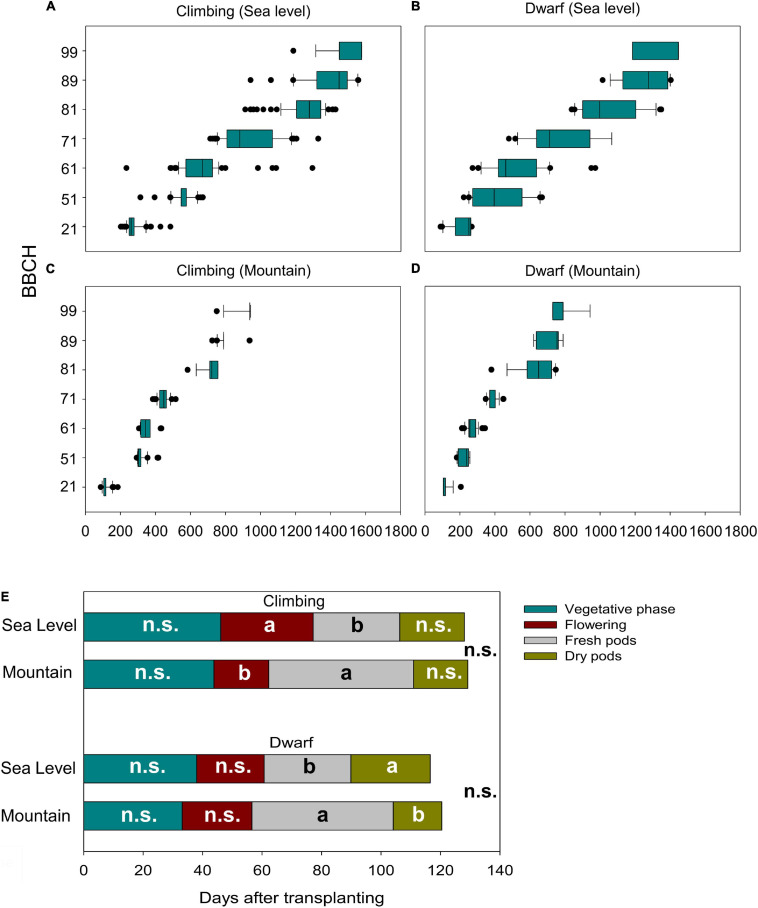
Relationship of the plant development (BBCH-scale) and the cumulative growing degree days (GDD) for climbing accessions at sea level **(A)** in the mountains **(C)** and dwarf accessions at the sea level **(B)** and in the mountains **(D)** and the effect of the environments on the length of development stages of climbing and dwarf accessions **(E)**. Different letters in the same stage indicate a significant difference (α = 0.05); n.s., not significant differences.

The cultivation environment played a dramatic role in affecting the phenological behavior of the beans. Both climbing (Figures 3A,C) and dwarf (Figures 3B,D) accessions required more than twice the total ΣGDD to complete their cycles at sea level than at the mountain level. The same was also observed when considering the GDD needed to reach the single phenological stages. At sea level, the variability among accessions of the GDD associated with the appearance of the single stage was much more pronounced. In particular, the highest variability was observed for the BBCH 61 stage, with ranges of 233–1295 GDD among climbing genotypes and 419–974 GDD among dwarf genotypes (Figures 3A,B). Instead, the same genotypes cultivated in the mountain environment showed the highest variability of GDD to reach the last phenological stages: from 724 to 938 GDD to reach BBCH 89 for climbing genotypes and 380–748 to reach BBCH 81 for dwarf genotypes (Figures 3C,D).

Considering the merged phenological stages (paragraph 2.5.1) for both climbing and dwarf genotypes, the duration of the vegetative phase, flowering phase, fresh pod period, dry pod period, and total cycle length were significantly influenced by the accessions but not influenced by the interaction between accessions and the environment of cultivation. The environment significantly affected only the flowering and fresh pod phases in climbing genotypes and fresh and dry pod phases in dwarf genotypes. Specifically, (i) the average flowering phase of climbing accessions at sea level was 31 days, whereas, in the mountains, it was significantly shorter, at 18 days; (ii) dwarf accessions had a significantly longer dry pod period at sea level (31 days) compared to on the mountain (18 days); (iii) both dwarf and climbing accessions had significantly longer fresh pod periods on the mountain (45 and 49 days, respectively) compared to at sea level (29 days for both); and (iv) although some phases were significantly different, the cultivation environment did not affect the total cycle length or climbing for dwarf genotypes (Figure 3E).

### Seed Production

**FIGURE 4:**
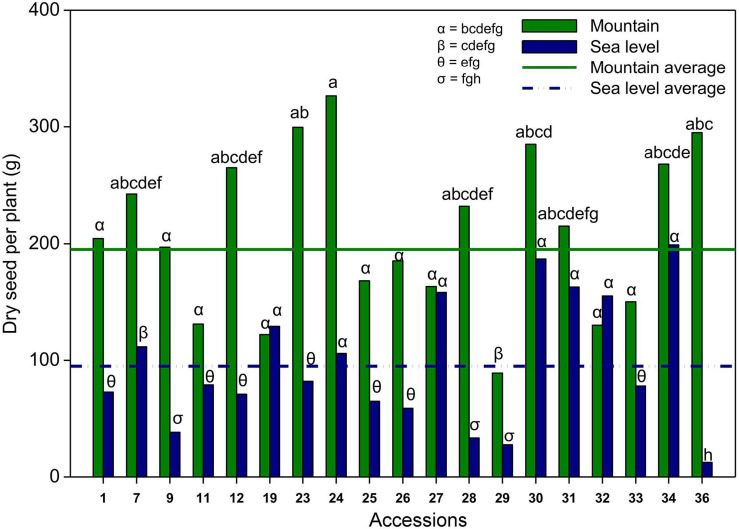
Average seed production per plant of each climbing accessions in the mountain (black bars) and at the sea level (gray bars), and average seed production in the mountain (solid line) and at the sea level (dash-dotted line). Different letters indicate a significant difference between compared groups. α = 0.05.

The interaction between the environment and accession significantly affected the seed production of climbing accessions (Table 7). The seed production of climbing accessions ranged from 12.4 (36 at sea level) to 326.4 g of seeds per plant (24 in the mountains). On average, climbing accessions produced approximately two times more seeds per plant on the mountain than at sea level. Of the 19 climbing accessions, only two produced more at sea level than at the mountain level; however, there was no significant difference. Accession 36 produced approximately 20 times more seeds in the mountains than at sea level (Figure 4).

**TABLE 7:** Analysis of variance (ANOVA) table with the effects of accession, environment, and the interaction of accession and environment on climbing and dwarf accessions phenology, seed production, seeds’ physical characteristics, and seeds’ nutraceutical properties.

	Climbing			Dwarf		
	Accession	Environment	Accession × Environment	Accession	Environment	Accession × Environment
	**Phenology**					
Vegetative phase	**	n.s.	n.s.	**	n.s.	n.s.
Flowering phase	***	***	n.s.	***	n.s.	n.s.
Fresh pods	***	***	n.s.	***	***	n.s.
Dry pods	***	n.s.	n.s.	***	***	n.s.
Total cycle length	***	n.s.	n.s.	**	n.s.	n.s.
	**Seed production**					
g of dry seeds per plant	***	**	**	***	n.s.	n.s.
	**Seeds’ physical characteristics**					
Length	***	**	**	***	**	n.s.
Width	***	***	***	***	**	n.s.
Thickness	*	***	***	***	**	n.s.
Weight of 100 seeds	***	***	***	***	***	n.s.
Volume of 10 seeds	**	***	***	***	*	n.s.
Density	***	***	n.s.	n.s.	**	n.s.
	**Nutraceutical properties**					
Total phenols	*	n.s.	n.s.	**	n.s.	n.s.
Antioxidant capacity	***	***	n.s.	***	***	n.s.
Protein	***	**	n.s.	***	n.s.	n.s.
Starch	***	n.s.	n.s.	***	**	n.s.
Essential amino acids	*	n.s.	n.s.	*	n.s.	n.s.
Non-essential amino acids	*	n.s.	n.s.	*	n.s.	n.s.

***p < 0.001; **p < 0.01; *p < 0.05; n.s., non-significant effect (*p* > 0.05).

Seed production was significantly different among dwarf accessions and was not affected by the environmental effects (Table 7). On average, the production of seeds of dwarf accessions ranged from 20.3 (accession 20) to 82.7 g of seeds per plant (accession 6). The dry seed production of accession 6 was significantly greater than those of accessions 17 (27.7 g) and 20 (20.3 g) (Supplementary Table 3).

### Seed Physical Characterization

The interaction between the environment and accession had a significant effect on the weight of 100 seeds of climbing accessions (Table 7). The weight of 100 seeds ranged from 25 g for accession 7 at sea level to 94 g for accession 33 on the mountain. Most of the 19 climbing accessions had 100-seed weights that were greater in the mountains than at sea level (Supplementary Table 4). Among dwarf accessions, the environment and the accession had a significant effect on the weight of 100 seeds (Table 7). In the mountainous environment, the weight of 100 seeds was significantly greater than that at sea level, and accessions 2 and 3 had the greatest weights of 100 seeds, whereas accession 17 had the lowest (Supplementary Table 3).

The length, width, and thickness of the seeds and the volume of 10 seeds of climbing accessions were significantly affected by the interaction between the environment and accession (Table 7). The volume of 10 seeds ranged from 2 (accession 7 in the mountain) to 7.7 mL (accession 32 at sea level). Seed lengths ranged from 10.5 (19 on the mountain) to 17.6 mm (32 on the mountain), widths ranged from 5.8 (12 on the mountain) to 12.2 mm (36 on the mountain), and thicknesses ranged from 4.7 (12 on the mountain) to 9.4 mm (36 on the mountain) (Supplementary Table 4). Among dwarf accessions, the environment had a significant effect on the volume of 10 seeds and on the seeds’ length, width, thickness, and density (Table 7), with these variables being significantly greater in seeds from the mountains compared to the seeds from sea level (Supplementary Table 3).

### Seed Nutraceutical Characterization

The total phenol contents of climbing and dwarf accessions were not significantly affected by the interaction between the environment and accession. There was a significant difference among the accessions, however, it was not significantly affected by the environment (Table 7). All accessions had a total phenol content greater than 3,000 mg GAE kg^–1^ of dry matter. Among the climbing accessions, accession 1 had the greatest total phenol content, with a significant difference compared to most of the other accessions, except accessions 12, 19, 24, 25, 30, 31, 32, 33, and 36. Accessions 7, 23, 29, and 34 had total phenol contents that were lower than 4,000 mg GAE kg^–1^ of dry matter. Among the dwarf accessions, the total phenol content of accession 8 seeds was significantly greater than that of accessions 6, 7, and 18 seeds. In addition to accession 8, accessions 2 and 3 also had a total phenol contents greater than 4,000 mg GAE kg^–1^ of dry matter (Table 8).

**TABLE 8:** Effects of the environments and of the accession on starch, protein, antioxidant capacity, and total phenols content of climbing and dwarf accessions.

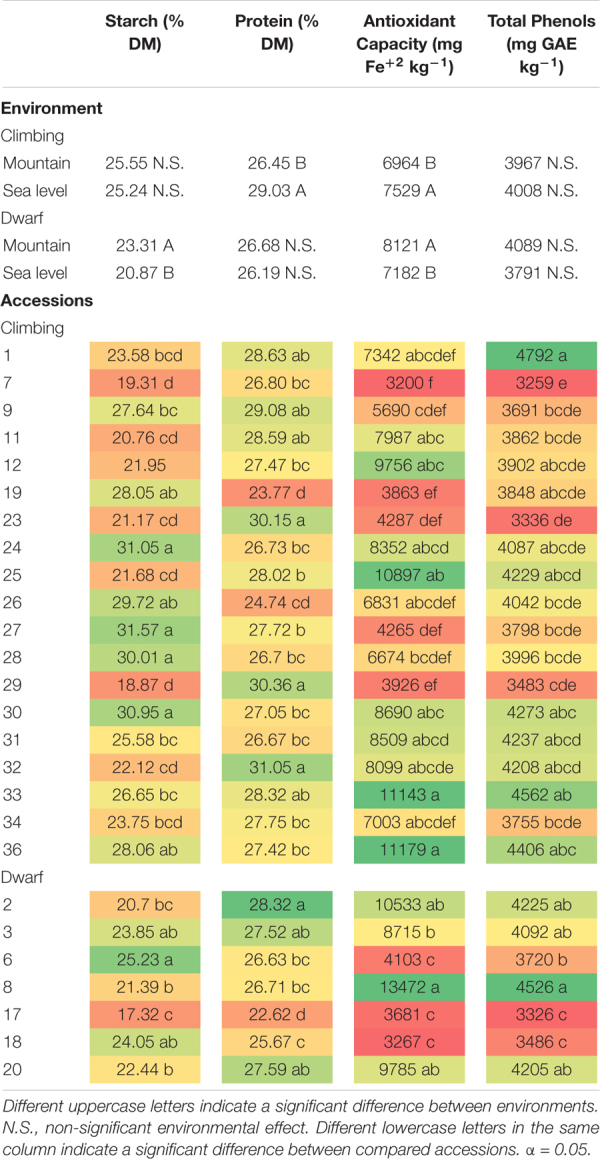

The antioxidant capacity of climbing and dwarf accessions was not significantly affected by the interaction between the environment and accession. However, there was a significant difference among the accessions and between the environments (Table 7). Most of the accessions’ antioxidant capacities were over 6,000 mg Fe^+2^ kg^–1^. Among the climbing accessions, those of accessions 25, 33, and 36 were over 10,000 mg Fe^+2^ kg^–1^, which were significantly greater contents than those of accessions 7, 9, 19, 23, 27, and 29, with antioxidant capacities lower than 6,000 mg Fe^+2^ kg^–1^. Among the dwarf accessions, the content of 8 was significantly greater than those of accessions 3, 6, 17, and 18. In addition to accession 8, which had a content greater than 13,000 mg Fe^+2^ kg^–1^, accession 2 also had an antioxidant capacity greater than 10,000 mg Fe^+2^ kg^–1^. The contents of accessions 6, 17, and 18 were lower than 4,000 mg Fe^+2^ kg^–1^. The antioxidant capacity was significantly greater at sea level for climbing accessions and in the mountains for dwarf accessions (Table 8).

The starch content of dwarf accessions was significantly affected by the environment (Table 7), and seeds cultivated in the mountains had a significantly greater starch content than those cultivated at sea level. Accession 6 had a significantly greater starch content than those of accessions 2, 8, 17, and 20 (Table 8). The environment did not affect the starch content of climbing accessions (Table 7). Accessions 24, 27, 28, and 30 had starch contents greater than 30%, whereas accessions 7 and 29 had starch contents lower than 20% (Table 8).

Accessions and the environment significantly affected the seed protein content of climbing accessions (Table 7), which was 9.75% greater at sea level than at mountain level. Accessions 23, 29, and 32 had protein contents greater than 30%, whereas accessions 19 and 26 had protein contents lower than 26% (Table 8). The environment did not have a significant effect on the protein content of dwarf accessions; however, there was a significant difference among accessions (Table 7). All dwarf accessions had protein contents lower than 30%, and accessions 17 and 18 had protein contents lower than 26% (Table 8).

The amino acid profile was not affected by the environment or by the interaction between the environment and accession (Table 7). In terms of essential amino acids, there was a significant difference in the contents of methionine and threonine of climbing accessions and in the threonine content of dwarf accessions. Among climbing accessions, in accessions 1 (321 mg 100 g^–1^) and 36 (310 mg 100 g^–1^), the methionine contents were significantly greater than those in accessions 12, 19, 26 (all 256 mg 100 g^–1^), and 7 (253 mg 100 g^–1^), and in accession 1, the threonine content (1,261 mg 100 g^–1^) was significantly greater than those of accessions 25 (899 mg 100 g^–1^) and 30 (911 mg 100 g^–1^). Among dwarf accessions, the threonine content of accession 2 (1,179 mg 100 g^–1^) was significantly greater than that of accession 20 (1,100 mg 100 g^–1^), which was significantly greater than those of accessions 3 (1,003 mg 100 g^–1^), 6 (1,013 mg 100 g^–1^), 17 (1023 mg 100 g^–1^), and 18 (1,045 mg 100 g^–1^) (Supplementary Table 5). For the nonessential amino acids, there were significant differences in tyrosine among climbing accessions and in serine and glycine among dwarf accessions. Among climbing accessions, the accession 1 tyrosine content (811 mg 100 g^–1^) was significantly greater than that of accession 26 (596 mg 100 g^–1^). Among dwarfs, the accession 6 serine content (1,334 mg 100 g^–1^) was significantly lower than those of accessions 2 (1,611 mg 100 g^–1^), 8 (1,549 mg 100 g^–1^), 17 (1,433 mg 100 g^–1^), and 20 (1,567 mg 100 g^–1^), and the glycine contents of accessions 2 (1,004 mg 100 g^–1^) and 20 (986 mg 100 g^–1^) were significantly greater than that of accession 3 (852 mg 100 g^–1^) (Supplementary Table 6).

### Principal Component Analysis (PCA), Correlations, and Constellation Clustering

**FIGURE 5:**
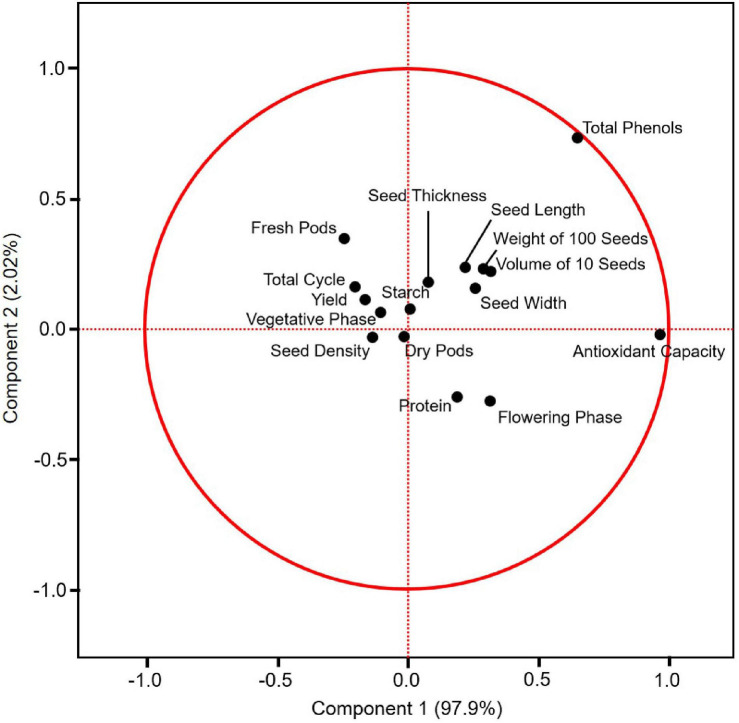
Principal component analysis (PCA) on the covariance of yield and the other variables of climbing accessions assessed in this study (i) and the clustered correlation between the variables (ii).

A principal component analysis (PCA) was performed with the data obtained in this study, except the amino acid profile. Principal component 1 (PC1) corresponded to 97.9% of the variability, and principal component 2 (PC2) corresponded to 2.02% of the variability. The weight of 100 seeds, the volume of 10 seeds, seed length, width, and thickness (all seed physical properties), and total phenol and starch contents were grouped in the quarters PC1+ and PC2+. The length of the flowering phase and the protein content were grouped in PC1+ and PC2−. The length of the dry pod period was in PC1- and PC2−. The yield, length of the total cycle, length of the fresh pod period and vegetative phase were grouped in the PC1− and PC2+ quarters (Figure 5).

The yield was significantly and positively correlated with the length of the fresh pod period (0.51, *p* < 0.0001) and significantly and negatively correlated with the length of the flowering phase (0.35, *p* < 0.0001) and the protein content (−0.42, *p* < 0.0001). The yield and the length of the fresh pod period were also significantly and positively correlated with the seed density, weight of 100 seeds, the volume of 10 seeds, and seed dimensions. The strongest correlations were between the weight of 100 seeds and the volume of 10 seeds and between these two variables and the seed dimensions. The antioxidant capacity and the total phenol content were also strongly correlated (0.77, *p* < 0.0001). The strongest negative correlations were between the protein content and seed density (−0.52, *p* < 0.0001) and between the protein content and starch content (−0.46, *p* < 0.0001) (Table 9).

**TABLE 9:** Correlation coefficients of the variables accessed in this study for climbing accessions, estimated by the REML (Restricted Maximum Likelihood) method.

	Yield	W100S	V10S	Density	Length	Width	Thick.	AC	TP	Prot.	Starch	Veg. Phase	Flow.	Fresh	Dry	Total Cycle
Yield	**1.00**															
W100S	**0.22**	**1.00**														
V10S	**0.14**	**0.98**	**1.00**													
Density	**0.34**	0.05	**−0.15**	**1.00**												
Length	**0.15**	**0.67**	**0.71**	**−0.20**	**1.00**											
Width	**0.22**	**0.80**	**0.78**	0.06	**0.52**	**1.00**										
Thick.	**0.32**	**0.70**	**0.65**	**0.19**	**0.34**	**0.76**	**1.00**									
AC	**−0.21**	**0.28**	**0.32**	**−0.15**	**0.23**	**0.20**	**0.06**	**1.00**								
TP	**−0.16**	**0.36**	**0.40**	**−0.20**	**0.22**	**0.36**	**0.15**	**0.77**	**1.00**							
Prot.	**−0.42**	**−**0.06	0.03	**−0.52**	**0.23**	**−0.28**	**−0.36**	**0.21**	0.05	**1.00**						
Starch	**0.23**	**0.26**	**0.23**	**0.17**	**−**0.09	**0.39**	**0.46**	0.01	**0.17**	**−0.46**	**1.00**					
Veg. Phase	**−0.22**	**−0.15**	**−0.14**	0.07	**−0.23**	0.05	0.02	**−**0.06	0.08	**−0.30**	**0.19**	**1.00**				
Flow.	**−0.35**	**−0.15**	**−0.10**	**−0.27**	**−0.18**	**−0.10**	**−0.16**	**0.39**	**0.20**	**0.33**	**−0.12**	**−**0.03	**1.00**			
Fresh	**0.51**	**0.24**	**0.16**	**0.44**	**0.18**	**0.30**	**0.35**	**−0.37**	**−0.16**	**−0.45**	0.07	**−0.10**	**−0.79**	**1.00**		
Dry	**−**0.01	0.06	**0.11**	**−0.32**	**0.20**	**−0.12**	**−0.11**	**−**0.09	**−0.16**	**0.24**	0.02	**−0.26**	**−0.20**	**−0.18**	**1.00**	
Total Cycle	**0.15**	**0.16**	**0.17**	**−0.09**	**0.16**	**0.20**	**0.19**	**−0.20**	**−0.14**	**−0.12**	**0.09**	0.08	**−0.15**	**0.14**	**0.67**	**1.00**

*W100S = weight of 100 seeds; V10S = volume of 10 seeds; Thick = seed thickness; AC = antioxidant capacity; TP = total phenols; Prot. = protein content; Veg. phase = length of the vegetative phase; Flow. = length of the flowering phase. Correlation coefficients bolded indicate a *p* value lower than 0.01.*

**FIGURE 6:**
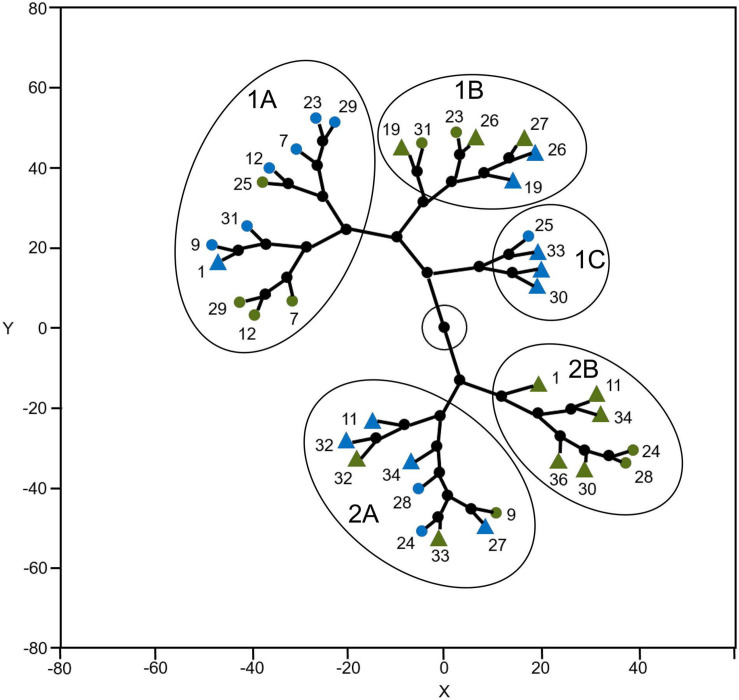
Constellation plot clustering climbing accessions and environment of cultivation based on the variables assessed in this study. M and green = cultivated in the mountain; S and blue = cultivated in the sea level; o = accession related to the Mesoamerican domestication center; Δ accession related to the Andean domestication center.

Considering the variables assessed in this study, climbing accessions were grouped according to the cultivation environment and the domestication center in constellation plot clustering. Five clusters were found: three when Y was positive (1A, 1B, and 1C) and two when Y was negative (2A and 2B). Cluster 1A is composed of 11 points, of which ten were accessions from the Mesoamerican domestication center, with six cultivated in the mountains and four cultivated at sea level, and one from the Andean center cultivated at sea level. Cluster 1B is composed of seven points, of which five are from the Andean domestication center (three cultivated in the mountain and two at sea level) and two are from the Mesoamerican domestication center cultivated in the mountains. Cluster 1C is composed of four points – three from the Andean domestication center and one from the Mesoamerican domestication center, all cultivated at sea level. Cluster 2A is composed of nine points, of which six are from the Andean domestication center (four cultivated at sea level and two in the mountains) and three are from the Mesoamerican domestication center (two cultivated at sea level and one in the mountains). Cluster 2B is composed of seven points, of which five are from the Andean domestication center and two are from the Mesoamerican domestication center, with all cultivated in the mountains (Figure 6).

## Discussion

Available historical records suggest that the common bean core collection we have studied is formed by neglected Venetian niche landraces locally maintained by farmers for several decades (*i.e.*, Venetian farmer’s local varieties) and old varieties genetically improved by breeders several decades ago using local materials (Italian breeder’s elite lineages). As a main finding, this core collection is represented by a large number of highly homozygous individuals and within-population homogeneous varieties. A considerable range of variation among populations is phenotypically detectable and genotypically verifiable, but between-population differentiation is also particularly evident and measurable for several distinctive plant and seed traits, likely as a consequence of both natural and human selection pressure.

### Molecular Characterization of Venetian Niche Landraces and Italian Elite Lineages and the Genetic Structure of the Core Collection as a Whole

The region of Veneto covers an area of 18,364 km^2^, of which 57% is a vast plain and 29% is a mountainous area composed of the Carnic Alps, Eastern Dolomites, and Venetian Prealps (Veneto Inside, 2020). This specific geographic formation allowed farmers or small farms and rural communities to grow beans in isolation for centuries. This study shows that over the years, new introductions and exchanges of different accessions have occurred from different domestication centers and origins. However, currently, based on visual characteristics, it is possible to identify dozens of landraces typical of that region. In addition to agronomic and nutraceutical categorization, one of the first goals of this study was the genetic and molecular characterization of a *P. vulgaris* core collection composed of local farmer varieties and elite breeder lineages using DNA markers. This approach was considered to be crucial for the genetic diversity estimation of potentially valuable bean germplasm resources to avoid any loss of genotypes/biotypes, to promote long-term conservation programs, and to allow commercial valorization of ancient bean varieties typical of the Veneto region, Italy.

The first SSR-based approach applied to a consistent number of samples (193 individuals belonging to 25 populations) highlighted a very high extent of homozygosity, which was in accordance with the autogamous reproduction system of this species characterized by a very low rate of occasional hybridization. Two important considerations are as follows: first, although homozygosity is linkage group-independent, its estimate was nearly equal to 100% and was found to be constant in the sequenced genes and amplified markers throughout the genome, and second, the set of expressed and neutral regions invested was shown to be representative of all the basic chromosomes, as confirmed by their genome and/or linkage map localization analysis.

The mean genetic similarity within each population was calculated based on all the pairwise comparisons among the accessions. The vast majority of populations scored a very high genetic similarity (>95%) and, hence, genetic uniformity, with 14 out of 25 varieties showing full genetic identity (*i.e.*, 100% genetic similarity estimates). This homogeneity can have different explanations, considering the different origins of the accessions. For the landraces (populations numbered from 23 to 36), the lack of variation within populations may be ascribed to the production system locally adopted by these niche plant materials in isolated geographical areas, which are often mountainous, limiting gene flow events among populations and preventing seed exchange among farmers. For the elite lineages (populations numbered from 1 to 20), the lack of variation within populations may be expected, as they are likely derived from single pure line selection methods to meet the genetic uniformity and stability requirements for commercial varieties. From the comparison among populations, four accessions (7, 23, 25, and 29) were highly differentiated from the rest, with a genetic similarity almost always below 70%. Interestingly, all the outliers previously mentioned have a Mesoamerican origin, and the majority (23, 25, and 29) are Venetian niche landraces. The Mesoamerican center is considered the first domestication center for the common bean, from which the Andean gene pool originated as a consequence of a strong bottleneck, and thus, the Mesoamerican gene pool is characterized by higher variability with the presence of uncommon genotypes. This peculiarity is also reflected in the core collection even after many generations of adaptation to the Italian environment. Overall, 56% of accessions were from the Andean center of origin, and 44% were from the Mesoamerican center. The proportion of Andean and Mesoamerican accessions in Veneto is close to that found by Angioi et al. (2010) in Europe: 67% Andean and 33% Mesoamerican. However, this proportion is slightly different when compared to their findings for Italian landraces: 75% Andean and 25% Mesoamerican. The data obtained in this study are also in agreement with Angioi et al. (2010) in terms of hybridization since their data showed that 44% of the European *P. vulgaris* landraces were derived from hybridization between Andean and Mesoamerican gene pools; however, in Spain and Italy, the distribution of hybrids was very low.

The SSR marker data were very useful for clustering the 193 samples initially selected into 41 distinct genotypes based on their genetic structures (allele/genotype diversity) and their genetic similarity estimates. This allowed us to construct a PCoA that led to some considerations. Generally, in populations represented by more than one genotype, it is possible to group the samples within narrow areas of the PCoA, meaning that even if not genetically identical, these populations possess a high degree of genetic homogeneity. Looking at the first dimension, a major division was clear between the elite lineages and the local varieties. This can be a result of the convergent evolution that led the local varieties to better adapt to the climatic and environmental conditions of the Veneto region and farmers’ cultivation methods, while the improved varieties are the results of breeders’ selection for their high yields and satisfying consumers’ preferences. The only misplacement is represented by population 1, possibly because this pure line was derived from local germplasm in recent times. Such a well-defined separation is not present in the second dimension, but samples of Andean origin showed the lowest values, while those of Mesoamerican origin showed the highest values. While such classification by ancestry origin was evident with the SNP marker data, upon using microsatellites, the result is not the same, thus highlighting the limits of this methodology based on neutral markers in reconstructing genetic relationships of more genetically distant accessions.

Microsatellite markers are known to be extremely useful for assessing the genetic similarity between individuals and populations of the same species and testing the genetic identity of varieties. However, they are not reliable for describing phylogenetic relationships. Based on the SSR marker-based genotyping results, 41 unique genotypes were selected to represent the genetic variability existing in the core collection under study and were further characterized through SNP variant-based haplotyping using specific genes as target regions.

The genetic variability of the core collection was evaluated using ten marker genes spanning a total of 6,533 nucleotides, where 48 SNPs and 8 INDELs were found. The SNP frequency was 1 every 136 bp, which is considerably high when compared to the average found in other legumes (1 every 233 bp in *Medicago trunculata* (Branca et al., 2011) and 1 every 588 bp in *Glycine max* (Lam et al., 2010)), meaning that the target regions chosen for this analysis are characterized by a particularly high polymorphism rate. As expected, introns were found to be less conserved, since mutations in these regions are silent and neutral, with no association with any phenotypic variation.

Considering only the SNP variants detected in exons, it is interesting to note that in 8 out of 17 cases, the mutation was nonsynonymous, determining an amino acid substitution. In these cases, the altered protein can be responsible for phenotypic variation. Our finding is particularly relevant since the genes chosen for the haplotyping analysis were selected based on their putative association with traits of agronomic interest.

Polymorphic nucleotides were actually arranged in a few unique combinations. A total of 21 multilocus haplotypes was detected for the marker genes used, with 7 in cluster A and 14 in cluster B. Fifteen of these SNP-derived haplotypes were encountered in less than 5% of the accessions, mainly in those attributable to Mesoamerican ancestry (cluster B). The presence of uncommon gene variants caused an increase in the genetic diversification of this subgroup, which may be exploited as a useful resource for the development of new varieties with unique characteristics and adapted to local conditions. These results are in agreement with the previous SSR marker-based analysis, which also highlighted the higher variability in the group with a Mesoamerican origin. Another aspect to take into account is the functionality of the allele associated with a specific haplotype, which implies that genetic mutations are translated into protein modifications. Nonfunctional haplotypes are useful to reconstruct the evolutionary history of an organism but will not be reflected in phenotypic variations. In total, 9 distinct functional haplotypes were present in the core collection – 3 present in cluster A and 8 in cluster B, with 2 shared between the two groups. It is thus possible that in cluster B, not only the genetic diversity but also the phenotypic diversity is higher. Selection within a wide gene pool has a higher probability of finding individuals with particular traits, conferring adaptability to mutable or extreme environments or resistance to pests and diseases. These characteristics are becoming increasingly valuable considering climate change and the growing demand for food that the world will face in the upcoming years.

The abundance of different genotypes of Mesoamerican origin was also evident in the UPGMA dendrogram, which was composed of branches grouping only 1 or 2 populations. One of the experimental lines (population 12), which were also selected based on uniformity criteria, was even composed of 5 genetically distinct genotypes. The subgroup with Andean origin was markedly different and much more uniform. In cluster A, 15 of the 22 genotypes were grouped and shared the same haplotype. Experimental lines and local varieties in some cases cannot be distinguished, and this may indicate that they are related and that pure lines were developed from those landraces or closely related materials.

Comparing the molecular tools used in this study, noncoding region-based SSR markers (neutral regions) revealed more variability in the Andean gene pool, whereas the coding region-based SNP markers (expressed regions) detected higher polymorphism in the Mesoamerican gene pool. One example is population 36 (“Maron”), for which the SSR analysis result was one of the most variable, while the subsequent haplotyping defined it as almost uniform. Moreover, SSR markers showed greater informativeness about the common bean typology – Venetian niche landraces or Italian lineage – compared to SNP markers, which otherwise demonstrated higher suitability for ancestral gene pool reconstruction (for details, see Figure 1).

The clear distinction between the Andean and Mesoamerican gene pools was not ensured since the genetic material used for this study can be geographically ascribed to northeastern Italy. Europe, in fact, is considered a secondary diversification center where gene flow occurred by spontaneous events of hybridization and introgression, but still, the distinction into two well-separated clusters calculated for the ancestry reconstruction of the analyzed samples is noteworthy.

Remarkable are the results obtained from the analysis of the intronic region of the shatterproof gene, where an extremely high abundance of polymorphisms was localized – totals of 10 SNPs and 3 INDELs were located in a 440 bp-long sequence. This marker gene has proven to be extremely predictive in distinguishing between Andean and Mesoamerican origin. The presence of intraspecific indels is also a particularly relevant and peculiar feature, which could be exploited for the development of cheap and fast molecular tools able to discriminate the gene pool of origin based only on the length of this fragment. It will be necessary to perform additional investigations with individuals of different geographical origins to confirm this hypothesis.

Thus, by using neutral SSR markers in combination with functional SNP markers, it was possible to unambiguously group the 25 *P. vulgaris* populations (“Semirampicante abruzzese”) into two clusters based on their gene pool of origin – either Andean or Mesoamerican. Genetic variability was distributed unequally across the two subgroups, as highlighted by haplotyping analysis, in which 16 out of the 23 multilocus haplotypes detected in the core collection were found within the Mesoamerican gene pool, while the Andean was much more homogeneous.

The molecular characterization provided useful information for the selection of pure lines among the analyzed populations, which can be combined with the nutraceutical characterization and the agronomic performance of these accessions in different Venetian environments. These are fundamental steps for the development of new varieties highly adaptable to local agronomic and climatic conditions and valuable for organic cultivation systems. Italian citizens value local food with a strong connection to the production area, increasing their value and demand in a microregion (Belletti et al., 2017), and another factor that can add value to these varieties is represented by the possibility of their genetic traceability along the whole supply chain using the described molecular tools to guarantee high-quality standards and to protect consumers and producers.

### Agronomic Performance of Varieties Under Organic Farming in Distinct Venetian Environments

The vegetative and flowering stages of dwarf accessions lasted approximately the same time in both locations, leading to the same timing of fresh and ripening pods. These accessions did not have a significant difference in production per plant when comparing both locations, producing, on average, approximately 55 g of dry seed per plant. Since all seven dwarf accessions are elite lines, it was expected that seed production would have a lower environmental effect due to its high phenotypic plasticity. The environment had a significant effect on the seed production of climbing accessions. The fresh pod stage is important in achieving greater yields since the seeds are filled and developed, having a direct impact on the yield. For climbing accessions, the length of the fresh pod period was positively and significantly correlated with the weight of 100 seeds (0.24, *p* < 0.01), the volume of 10 seeds (0.16, *p* < 0.01), seed density (0.44, *p* < 0.01), and seed length (0.18, *p* < 0.01), width (0.30, *p* < 0.01), and thickness (0.35, *p* < 0.01), indicating that the longer fresh pod period in the mountain produced more seeds that were heavier and larger, leading to a strong positive correlation of the fresh pod period with the yield (0.51, *p* < 0.01). On the other hand, the flowering phase was 13 days shorter in the mountain and negatively related to the yield (−0.35, *p* < 0.01) and to the other seed physical characteristics.

The cumulative growing degree days (GDD) is a tool widely used to predict crop development through different linear and nonlinear methods (Zhou and Wang, 2018). In this study, the same accessions cultivated in two environments with dramatically different temperatures closed the productive cycle with very different thermal sums: 1,800 ΣGDD at Legnaro and 600 at Asiago. Surprisingly, the duration of the growing cycle was quite similar in the two environments, suggesting that the thermal sum is not an adequate tool with which to predict phenology. In fact, the assumption of the thermal sum is that “the higher the cumulative temperature, the faster the phenological development.”

Most likely, the discrepancy between the assumptions and observations is because we used the simplest version of the formula to calculate the GDD, and we used the most common base temperature, 10°C, for common beans (Jenni et al., 2000). However, each accession may have its own minimum base temperature. Jenni et al. (2000) showed that by changing the minimum base temperature from 10 to 0°C, bean cultivars would increase their growing degree days from sowing to maturity from approximately 600 to approximately 1,200. In addition, the maximum temperature above which plant growth is zero could influence the GDD calculations. Indeed, most of the analyzed genotypes are adapted to the mountainous environment, and they probably do not take advantage of warmer temperatures. However, the optimal temperature for the common bean has been reported to be in the range between 28 and 34°C (White and Montes-R, 1993; Yan and Hunt, 1999), and during the experimental period, the average temperature was never above this threshold. We can hypothesize that a calculation of the thermal sum based on hourly temperatures would be more adequate to predict phenology. Historical meteorological data have shown that both locations, Asiago and Legnaro, have become warmer over the years. Since most of these landraces are adapted to milder weather, predicting the temperature variation effects is crucial for their successful cultivation.

Considering a spacing of 0.5 m × 0.5 m for each climbing plant in a typical Veneto bean production system, there are approximately 4,000 plants per hectare. Thus, the seed yield in the mountain was approximately 840 kg of dry beans per hectare for the landraces. This value was reduced to 380 kg per hectare at sea level. Accessions 23, 24, 30, and 36 (Gialet, Posenati, Seclé, and Maron) achieved greater yields in Asiago, at 1,200, 1,300, 1,080, and 1,080 kg per hectare, respectively. In 2004, Piergiovanni et al. called attention to the risk of the disappearance of these typical Veneto ecotypes, highlighting that in terms of yield, landraces such as Gialet do not reach the quantities needed to compete with Lamon beans (4,000 kg ha^–1^ vs. <2,000 kg ha^–1^), which is another typical landrace with the protected geographic indication (PGI) from the region of Belluno in Veneto (Italian Made, 2020). However, due to the diversity of the characteristics of the seeds and the quality, it is common to find that these ecotypes are sold in farmers’ markets throughout Veneto at prices higher than common commercial beans. The Gialet from Belluno was sold from 18 to 25 euros per kg, and the Lamon bean IGP was sold from 20 to 25 euros per kg in 2017, according to the Treviso-Belluno Chamber of Commerce (Camera di Commercio Treviso-Belluno, 2017). However, most of these niche landraces are commercialized in very specific and small areas and are likely to be replaced by landraces with higher prices and better yields (Ranalli and Parisi, 2018). This fact increases the importance of conserving them in germplasm and knowing and describing their genetic, agronomic, morphologic, and nutraceutical characteristics. Additionally, the cultivation of beans is a cultural tradition and is often seen in small gardens in the backs of houses, and often, the exchange or commercialization occurs between friends or known neighbors. This is one of the reasons why all Veneto niche landraces are climbing and have indeterminate growth. These characteristics facilitate manual harvesting and allow staggered harvesting for weeks or even months of fresh beans.

The high genetic and phenotypic diversity of the Venetian niche landraces and other accessions assessed in the VALEBIO Project is also present in the morphologic characteristics of the seeds and the structure of the seeds. In addition to different shapes and colors, seed dimensions and weights also varied among the accessions. The seed length ranged from 10.2 to 17.4 mm, the width ranged from 5.2 to 11.3 mm, and the thickness ranged from 4.9 to 8.3 mm among the 26 accessions that completed their cycles in both locations. The weight of 100 seeds ranged from 24.68 to 95.02 g, and the volume of 100 seeds ranged from 15 to 77 mL. The seed dimensions of the accessions assessed in this study have a greater range than those described by Giurcă (2009), and the weight of 100 seeds is close to the weight of Venetian agroecosystems presented by Piergiovanni et al. (2004). In 2019, Nicoletto et al. assessed the effect of different Venetian environments on the seed properties of two Venetian landraces and did not find significant effects of the environment on the 100-seed weight, seed volume, or seed density. In this study, more accessions were assessed, and a significant difference between the different environments was observed for the weight of 100 seeds, seed length, seed thickness, seed width, seed volume, and seed density. The seeds from the mountain had greater weight, length, width, thickness, and density.

### Nutraceutical Characterization of Seeds

Nicoletto et al. (2019) found a significant effect of the environment on the total phenols and total antioxidant capacity of two Venetian niche landraces. Of the accessions assessed in this study, a significant difference was found between both locations. The differences among accessions in the total phenol content and antioxidant capacity were significant. Akond et al. (2011) assessed the total polyphenols and antioxidant capacity of 29 common beans from Mexico, the United States, Brazil, and India. The total phenols ranged from 5,870 to 14,140 mg of GAE kg^–1^. Garretson et al. (2018) assessed the total phenols and antioxidant capacity of five raw heirloom bean seeds, and the amounts ranged from 4,800 to 9,600 mg of GAE kg^–1^. In this study, the total phenols ranged from 3,900 to 4,791 mg of GAE kg^–1^ among the accessions in both locations, indicating that Italian beans have a lower total phenol content than those cultivated in the Americas. On the other hand, Nicoletto et al. (2019) assessed the Venetian niche landraces Lingua di Fuoco and Gialet (accessions 8 and 23 in this study) and found total phenol contents of 1,240 and 1,047 mg GAE kg^–1^, respectively, which were much lower than those found in this study, at 3,335 and 3,468 mg GAE kg^–1^, respectively. This study only performed the nutraceutical characterization of dry seeds, which is not how the beans are normally consumed. Soaking the bean seeds makes the cells softer, which can solubilize bound polyphenols that can be absorbed by the water, reducing the contents in the beans, as shown by Boateng et al. (2008). Rocha-Guzmán et al. (2007) found that cooking beans under pressure can reduce the phenolic content in the seed coat by 90%, and their results are in agreement with Barroga et al. (1985), who boiled another legume, the mung bean (*Vigna radiata*), for 30 min and found that it reduced the phenolic content by 73%. Other authors, however, have shown an increase in the content of phenols (Fernández et al., 1982; Vidal-Valverde et al., 1994).

When compared to other legumes, Gutiérrez-Uribe et al. (2011) found lower values for the whole seed of cowpeas, at 755.7 mg GAE kg^–1^. Kumar et al. (2010) compared the total phenol contents of soybean accessions of different colors: yellow, green, and black. They found lower values in the green and yellow accessions, ranging from 960 to 2,890 mg GAE kg^–1^. However, the range found in this study was within the range found for soybean accessions with black seeds, from 810 to 5,890 mg GAE kg^–1^. The antioxidant capacity ranged from 3,267 mg Fe^+2^ kg^–1^ to 13,472 mg Fe^+2^ kg^–1^. The total antioxidant capacities of Lingua di Fuoco and Gialet assessed by Nicoletto et al. (2019) were 3,482 and 2,133 mg Fe^+2^ kg^–1^, respectively, which were also lower than the contents obtained for the same accessions in this study, at 4,287 and 7,986 mg Fe^+2^ kg^–1^, respectively. The total phenol content and antioxidant capacity had a strong significant positive correlation (0.77, *p* < 0.01), similar to the correlation obtained by Mastura et al. (2017) (0.77, *p* < 0.01) and Chávez-Mendoza et al. (2019) (0.88, *p* < 0.01). Accessions 6, 7, 17, and 18 had low contents of total phenols and antioxidant capacities, and their seed colors were white or pale yellowish. Accessions 33, 34, 36, and 8 had the greatest contents of total phenols and antioxidant capacities. The seed colors of these accessions were light brown/dark brown, golden brown, brown, and purple/white, respectively. Thus, this study confirms the relationship between the seed coat color and the seed total phenols and antioxidant capacity stated by Díaz-Batalla et al. (2006); Oroian and Escriche (2015), and Ombra et al. (2016).

Guzmán-Maldonado et al. (2000) characterized 70 wild and weedy common beans from Durango and Jalisco (Mexico) and found that the protein content ranged from 18.0 to 33.0%. The protein content of 59 accessions from North Spain and five commercial cultivars ranged from 19.3 to 25.2% (Escribano et al., 1997), and that of 73 South Brazil landraces ranged from 19.0 to 31.0% (Pereira et al., 2016). The protein content of the Italian common beans assessed in this study ranged in agreement with the studies mentioned before, from 19.35 to 33.55%. The environment had a positive effect on the protein content of climbing varieties. On average, seeds from sea level had a protein content of 29.03%, while seeds from the mountain had a protein content of 26.45%, with a decrease in the protein content between the two environments by 9.75%. This difference in the protein content can be explained by the negative and significant correlation with the yield (−0.42, *p* < 0.01), with the accessions cultivated in the mountain producing more seeds but lower protein contents. A similar negative correlation between the yield and protein was found in other recent studies (Kazai et al., 2019; Bulyaba et al., 2020). The negative correlation between yield and protein content can be explained by the dilution effect of a greater carbohydrate content and reduced nitrogen in the endosperm (Davis et al., 2004; Sica et al., 2021). The starch content had a wider range than the protein content, from 14.12 to 35.25%, and the environment had a significant effect on the starch content of the dwarf accessions, which was significantly greater in Asiago. The accession and the environment had a low effect on the contents of most amino acids.

### Venetian Niche Landraces With Potential to Become Commercial Varieties

Venetian niche landraces showed a strong connection to the mountainous environment, producing, on average, two times more on the mountain than at sea level. Based on their genetic characteristics, agronomic performance in one or both environments, and nutraceutical properties, five Venetian niche landraces can be highlighted and considered to have greater potential to become commercial varieties. Gialet, which means yellowish in the Veneto dialect, had great agronomic performance in both locations and high protein content. Posenati, from Posina in Vicenza, Veneto, had great agronomic performance in both locations and had high total phenols and antioxidant capacity. Although its seeds are not colorful, they are visually similar to the traditional Lamon bean, so it can have commercial appeal in the region. Seclè had a great performance in both locations and a high content of total phenols and antioxidant activity. Its purple seeds are visually appealing and can increase its commercial value. D’oro, meaning of gold, had a great performance in both locations, and its seeds have good commercial characteristics. Maron, which is brown, had a good performance in the mountain, producing 20 times more compared to at sea level; thus, it would be a commercial variety recommended to be cultivated in the mountains. Maron also has high contents of total phenols and antioxidant activity, and its seed characteristics can increase its commercial value. Sica et al. (2021) assessed the effect of drought on the yield and nutraceutical properties of these five landraces and found out that Gialet’s yield was not significantly affected by drought and Seclè’s yield increased under drought conditions during the vegetative phase. In addition, the zinc content of Gialet’s and Maron’s seeds was equal to about 60 mg kg^–1^, which is two times greater than the average content scored by commercial varieties.

Therefore, this study provides original information that allows not only the conservation of this genetic material from Venetian niche landraces and Italian elite lineages, but also the commercial valorization of this genetic material with a strong connection to the region where they have been cultivated for centuries, thereby allowing the selection of landraces with good agronomic performance and high nutraceutical value, which may become commercial varieties with high added value. Italian citizens value local food with a strong connection to the production area, thus increasing their value and demand in a microregion. Another factor that can add value to these varieties is represented by the possibility of their genetic traceability along the whole supply chain using the described molecular tools to guarantee high-quality standards and to protect consumers and producers.

## Data Availability Statement

The original contributions presented in the study are included in the article/Supplementary Material, further inquiries can be directed to the corresponding author/s.

## Author Contributions

GB, PSa, MB, CN, and CM contributed to conceptualization. FS, DB, CM, and CN contributed to formal analysis. PSi, FS, DB, CM, and CN contributed to methodology, contributed to investigation, and contributed to writing – original draft preparation. GB, PSa, and MB contributed to resources and contributed to supervision. All authors contributed to writing – review and editing and have read and agreed to the published version of the manuscript. GB and CN contributed to visualization. GB contributed to project administration.

## Conflict of Interest

The authors declare that the research was conducted in the absence of any commercial or financial relationships that could be construed as a potential conflict of interest.

## Footnotes

1 https://phytozome.jgi.doe.gov

2 https://www.ncbi.nlm.nih.gov/tools/primer-blast

## References

[B1] Adler-Nissen J. (1986). *Enzymatic Hydrolisis of Food Protein.* New York: Elsevier.

[B2] Akond A. S. M. G. M. Khandaker L. Berthold J. Gates L. Peters K. Delong H. (2011). Anthocyanin, total polyphenols and antioxidant activity of common bean. *Am. J. Food Technol.* 6 385–394. 10.3923/ajft.2011.385.394

[B3] Albala K. (2007). “Phaseolus vulgaris: mexico and the world,” in *Beans: A History* (London, UK: Bloomsbury Academic), 240.

[B4] Angioi S. A. Rau D. Attene G. Nanni L. Bellucci E. Logozzo G. (2010). Beans in Europe: origin and structure of the European landraces of *Phaseolus vulgaris* L. *Theor. Appl. Genet.* 121 829–843. 10.1007/s00122-010-1353-2 20490446

[B5] Ariani A. Berny Mier y Teran J. C. Gepts P. (2016). Genome-wide identification of SNPs and copy number variation in common bean (*Phaseolus vulgaris* L.) using genotyping-by-sequencing (GBS). *Mol. Breed.* 36 1–11. 10.1007/s11032-016-0512-9

[B6] Ariani A. Mier Teran J. C. B. Gepts P. (2018). Spatial and temporal scales of range expansion in wild *Phaseolus vulgaris*. *Mol. Biol. Evol.* 35 119–131. 10.1093/molbev/msx273 29069389 PMC5850745

[B7] Arnoldi A. Zanoni C. Lammi C. Boschin G. (2015). The role of grain legumes in the prevention of hypercholesterolemia and hypertension. *Crit. Rev. Plant Sci.* 34 144–168. 10.1080/07352689.2014.897908

[B8] Barroga C. F. Laurena A. C. Mendoza E. M. T. (1985). Polyphenols in mung bean (*Vigna radiata* (L.) Wilczek): determination and removal. *J. Agric. Food Chem.* 33 1006–1009. 10.1021/jf00065a056

[B9] Beebe S. Rengifo J. Gaitan E. Duque M. C. Tohme J. (2001). Diversity and origin of Andean landraces of common bean. *Crop Sci.* 41 854–862. 10.2135/cropsci2001.413854x

[B10] Beebe S. Skroch P. W. Tohme J. Duque M. C. Pedraza F. Nienhuis J. (2000). Structure of genetic diversity among common bean landraces of middle american origin based on correspondence analysis of RAPD. *Crop Sci.* 40 264–273. 10.2135/cropsci2000.401264x

[B11] Belletti G. Marescotti A. Brazzini A. (2017). “Old world case study: the role of protected geographical indications to foster rural development dynamics: the case of sorana bean PGI,” in *The Importance of Place: Geographical Indications as a Tool for Local and Regional Development*, eds van Caenegem W. Cleary J. (Cham: Springer), 253–276. 10.1007/978-3-319-53073-4_10

[B12] Benzie I. F. F. Strain J. J. (1996). The ferric reducing ability of plasma (FRAP) as a measure of “antioxidant power”: the FRAP assay. *Anal. Biochem.* 239 70–76. 10.1006/abio.1996.0292 8660627

[B13] Bioline L. U. (2020). *PCR, qPCR & NGS Reagents | Bioline | Meridian Bioscience.* Available online at: https://www.bioline.com/ (accessed November 15, 2020)

[B14] Bishnoi S. Khetarpaul N. (1993). Variability in physico-chemical properties and nutrient composition of different pea cultivars. *Food Chem.* 47 371–373. 10.1016/0308-8146(93)90179-J

[B15] Blair M. W. Torres M. M. Giraldo M. C. Pedraza F. (2009). Development and diversity of andean-derived, gene-based microsatellites for common bean (*Phaseolus vulgaris* L.). *BMC Plant Biol.* 9:100. 10.1186/1471-2229-9-100 19646251 PMC3091531

[B16] Boateng J. Verghese M. Walker L. T. Ogutu S. (2008). Effect of processing on antioxidant contents in selected dry beans (*Phaseolus* spp. L.). *LWT Food Sci. Technol.* 41 1541–1547. 10.1016/j.lwt.2007.11.025

[B17] Branca A. Paape T. D. Zhou P. Briskine R. Farmer A. D. Mudge J. (2011). Whole-genome nucleotide diversity, recombination, and linkage disequilibrium in the model legume *Medicago truncatula*. *Proc. Natl. Acad. Sci. U.S.A.* 108 E864–E870. 10.1073/pnas.1104032108 21949378 PMC3198318

[B18] Bulyaba R. Winham D. M. Lenssen A. W. Moore K. J. Kelly J. D. Brick M. A. (2020). Genotype by location effects on yield and seed nutrient composition of common bean. *Agronomy* 10:347. 10.3390/agronomy10030347

[B19] Calles T. (2016). Preface to special issue on leguminous pulses. *Plant Cell Tissue Organ Cult.* 127 541–542. 10.1007/s11240-016-1146-7

[B20] Camera di Commercio Treviso-Belluno (2017). *Indicazioni di Mercato Sui Prezzi Medi dei Prodotti Tipici Bellunesi. 1.* Available online at: http://webcache.googleusercontent.com/search?q=cache:FNHTLhECyJ0J:www.bl.camcom.it/UserFile/File/Statistica_Protesti/listino%2520prodotti%2520tipici%2520bellunesi/06_giugno%25202017(1).pdf+&cd=2&hl=pt-BR&ct=clnk&gl=us (accessed June 8, 2020)

[B21] Chávez-Mendoza C. Hernández-Figueroa K. I. Sánchez E. (2019). Antioxidant capacity and phytonutrient content in the seed coat and cotyledon of common beans (*Phaseolus vulgaris* L.) from various regions in Mexico. *Antioxidants* 8:5. 10.3390/antiox8010005 30585238 PMC6356214

[B22] Dal Ferro N. Borin M. (2017). Environment, agro-system and quality of food production in Italy. *Ital. J. Agron.* 12. 10.4081/ija.2017.793

[B23] Davis D. R. Epp M. D. Riordan H. D. (2004). Changes in USDA food composition data for 43 garden crops, 1950 to 1999. *J. Am. Coll. Nutr.* 23 669–682. 10.1080/07315724.2004.10719409 15637215

[B24] Díaz L. M. Blair M. W. (2006). Race structure within the Mesoamerican gene pool of common bean (*Phaseolus vulgaris* L.) as determined by microsatellite markers. *Theor. Appl. Genet.* 114 143–154. 10.1007/s00122-006-0417-9 17047911

[B25] Díaz-Batalla L. Widholm J. M. Fahey G. C. Castaño-Tostado E. Paredes-López O. (2006). Chemical components with health implications in wild and cultivated Mexican common bean seeds (*Phaseolus vulgaris* L.). *J. Agric. Food Chem.* 54 2045–2052. 10.1021/jf051706l 16536573

[B26] Escribano M. R. Santalla M. De Ron A. M. (1997). Genetic diversity in pod and seed quality traits of common bean populations from northwestern Spain. *Euphytica* 93 71–81. 10.1023/A:1002908224793

[B27] Evanno G. Regnaut S. Goudet J. (2005). Detecting the number of clusters of individuals using the software structure: a simulation study. *Mol. Ecol.* 14 2611–2620. 10.1111/j.1365-294X.2005.02553.x 15969739

[B28] Fernández R. Elís L. G. Braham J. E. Bressani R. (1982). Trypsin inhibitors and hemagglutinins in beans (*Phaseolus vulgaris*) and their relationship with the content of tannins and associated polyphenols. *J. Agric. Food Chem.* 30 734–739. 10.1021/jf00112a027

[B29] Garretson L. Tyl C. Marti A. (2018). Effect of processing on antioxidant activity, total phenols, and total flavonoids of pigmented heirloom beans. *J. Food Qual.* 2018 1–6. 10.1155/2018/7836745

[B30] Geneious (2020). *Geneious Bioinformatics Software for Sequence Data Analysis.* Available online at: https://www.geneious.com/ (accessed October 28, 2020)

[B31] Giurcă D. M. (2009). Morphological and phenological differences between the two species of the Phaselous genus (*Phaseolus vulgaris* and *Phaseolus coccineus*). *Cercet. Agron. Mold.* XLII, 39–45.

[B32] Goretti D. Bitocchi E. Bellucci E. Rodriguez M. Rau D. Gioia T. (2014). Development of single nucleotide polymorphisms in *Phaseolus vulgaris* and related *Phaseolus* spp. *Mol. Breed.* 33 531–544. 10.1007/s11032-013-9970-5

[B33] Gutiérrez-Uribe J. A. Romo-Lopez I. Serna-Saldívar S. O. (2011). Phenolic composition and mammary cancer cell inhibition of extracts of whole cowpeas (*Vigna unguiculata*) and its anatomical parts. *J. Funct. Foods* 3 290–297. 10.1016/j.jff.2011.05.004

[B34] Guzmán-Maldonado S. H. Acosta-Gallegos J. Paredes-López O. (2000). Protein and mineral content of a novel collection of wild and weedy common bean (*Phaseolus vulgaris* L). *J. Sci. Food Agric.* 80 1874–1881. 10.1002/1097-0010(200010)80:13<1874::AID-JSFA722<3.0.CO;2-X

[B35] Hidalgo R. (1988). “The phaseolus world collection,” in *Genetic Resources of Phaseolus Beans*, ed. Gepts P. (Dordrecht: Springer), 67–90. 10.1007/978-94-009-2786-5_4

[B36] Hjalmarsson S. Akesson R. (1983). Modern kjeldahl procedure. *Int. Lab.* 3 70–76.

[B37] Italian Made (2020). *Italian Made PDO/PGI.* Available online at: http://www.italianmade.com/usa/pdo-pgi/ (accessed June 1, 2020)

[B38] Jenni S. Bourgeois G. Laurence H. Roy G. Tremblay N. (2000). Improving the prediction of processing bean maturity based on the growing-degree day approach. *Am. Soc. Hortic. Sci.* 35 1234–1237. 10.21273/hortsci.35.7.1234

[B39] Johns M. A. Skroch P. W. Nienhuis J. Hinrichsen P. Bascur G. Muñoz-Schick C. (1997). Gene pool classification of common bean landraces from chile based on RAPD and morphological data. *Crop Sci.* 37 605–613. 10.2135/cropsci1997.0011183X003700020049x

[B40] Jones A. (1999). *Phaseoulus Beans: Post-Harvest Operations.* Quebec, QC: FAO, 1–25.

[B41] Kazai P. Noulas C. Khah E. Vlachostergios D. (2019). Yield and seed quality parameters of common bean cultivars grown under water and heat stress field conditions. *AIMS Agric. Food* 4 285–302. 10.3934/agrfood.2019.2.285

[B42] Kumar S. Banks T. W. Cloutier S. (2012). SNP discovery through next-generation sequencing and its applications. *Int. J. Plant Genomics* 2012:831460. 10.1155/2012/831460 23227038 PMC3512287

[B43] Kumar S. Stecher G. Li M. Knyaz C. Tamura K. (2018). MEGA X: molecular evolutionary genetics analysis across computing platforms. *Mol. Biol. Evol.* 35 1547–1549. 10.1093/molbev/msy096 29722887 PMC5967553

[B44] Kumar V. Rani A. Dixit A. K. Pratap D. Bhatnagar D. (2010). A comparative assessment of total phenolic content, ferric reducing-anti-oxidative power, free radical-scavenging activity, vitamin C and isoflavones content in soybean with varying seed coat colour. *Food Res. Int.* 43 323–328. 10.1016/j.foodres.2009.10.019

[B45] Kuzay S. Hamilton-Conaty P. Palkovic A. Gepts P. (2020). Is the USDA core collection of common bean representative of genetic diversity of the species, as assessed by SNP diversity? *Crop Sci.* 60 1398–1414. 10.1002/csc2.20032

[B46] Lam H. M. Xu X. Liu X. Chen W. Yang G. Wong F. L. (2010). Resequencing of 31 wild and cultivated soybean genomes identifies patterns of genetic diversity and selection. *Nat. Genet.* 42 1053–1059. 10.1038/ng.715 21076406

[B47] Mastura H. Hasnah Y. Dang H. (2017). Total phenolic content and antioxidant capacity of beans: organic vs inorganic abstract. *Int. Food Res. J.* 24 510–517.

[B48] McConnell M. Mamidi S. Lee R. Chikara S. Rossi M. Papa R. (2010). Syntenic relationships among legumes revealed using a gene-based genetic linkage map of common bean (*Phaseolus vulgaris* L.). *Theor. Appl. Genet.* 121 1103–1116. 10.1007/s00122-010-1375-9 20607211

[B49] Meier U. (2018). *Growth Stages of Mono-and Dicotyledonous Plants: BBCH Monograph.* Quedlinburg: Open Agrar Repositorium.

[B50] Nanni L. Bitocchi E. Bellucci E. Rossi M. Rau D. Attene G. (2011). Nucleotide diversity of a genomic sequence similar to SHATTERPROOF (PvSHP1) in domesticated and wild common bean (*Phaseolus vulgaris* L.). *Theor. Appl. Genet.* 123 1341–1357. 10.1007/s00122-011-1671-z 21830108

[B51] Nei M. (1987) *Molecular Evolutionary Genetics.* New York, NY: Columbia University Press 512.

[B52] Nelson M. E. Hamm M. W. Hu F. B. Abrams S. A. Griffin T. S. (2016). Alignment of healthy dietary patterns and environmental sustainability: a systematic review. *Adv. Nutr.* 7 1005–1025. 10.3945/an.116.012567 28140320 PMC5105037

[B53] Nicolè S. Erickson D. L. Ambrosi D. Bellucci E. Lucchin M. Papa R. (2011). Biodiversity studies in Phaseolus species by DNA barcoding. *Genome* 54 529–545. 10.1139/g11-018 21777058

[B54] Nicoletto C. Zanin G. Sambo P. Dalla Costa L. (2019). Quality assessment of typical common bean genotypes cultivated in temperate climate conditions and different growth locations. *Sci. Hortic.* 256:108599. 10.1016/j.scienta.2019.108599

[B55] Ombra M. N. D’acierno A. Nazzaro F. Riccardi R. Spigno P. Zaccardelli M. (2016). Phenolic composition and antioxidant and antiproliferative activities of the extracts of twelve common bean (*Phaseolus vulgaris* L.) endemic ecotypes of southern Italy before and after cooking. *Oxid. Med. Cell. Longev.* 2016 1–12. 10.1155/2016/1398298 28105248 PMC5220516

[B56] Oroian M. Escriche I. (2015). Antioxidants: characterization, natural sources, extraction and analysis. *Food Res. Int.* 74 10–36. 10.1016/j.foodres.2015.04.018 28411973

[B57] Pallottini L. Garcia E. Kami J. Barcaccia G. Gepts P. (2004). The genetic anatomy of a patented yellow bean. *Crop Sci.* 44 968–977. 10.2135/cropsci2004.9680

[B58] Palumbo F. Barcaccia G. (2018). “Critical aspects on the use of microsatellites markers for assessing genetic identity of crop plant varieties and authenticity of their food derivatives,” in *Rediscovery of Landraces as a Resource for the Future*, ed. Grillo O. (London: IntechOpen), 129–160.

[B59] Palumbo F. Galla G. Barcaccia G. (2017a). Developing a molecular identification assay of old landraces for the genetic authentication of typical agro-food products: the case study of the barley “agordino”. *Food Technol. Biotechnol.* 55 29–39. 10.17113/ftb.55.01.17.4858 28559731 PMC5434372

[B60] Palumbo F. Galla G. Martinez-Bello L. Barcaccia G. (2017b). Venetian local corn (*Zea mays* L) germplasm?: disclosing the genetic anatomy of old landraces suited for typical cornmeal mush production. *Diversity* 9:32. 10.3390/d9030032

[B61] Pereira T. Coelhor M. M. C. Bogo A. Guidolin A. Miquelluti D. J. (2016). Long time no see – rediscovery of peculiar ephemeral fern *Anogramma leptophylla* (L.) link in croatia. *Acta Bot. Croat.* 76 91–94. 10.1515/botcro-2016-0021

[B62] Piergiovanni A. R. Laghetti G. (1999). The common bean landraces from Basilicata (Southern Italy): an example of integrated approach applied to genetic resources management. *Genet. Resour. Crop Evol.* 46 47–52. 10.1023/A:1008641731573

[B63] Piergiovanni A. R. Lioi L. (2010). Italian common bean landraces: history, genetic diversity and seed quality. *Diversity* 2 837–862. 10.3390/d2060837

[B64] Piergiovanni A. R. Cerbino D. Brandi M. (2000). The common bean populations from Basilicata (Southern Italy). An evaluation of their variation. *Genet. Resour. Crop Evol.* 47 489–495. 10.1023/A:1008719105895

[B65] Piergiovanni A. R. Taranto G. Losavio F. P. Sanson S. (2004). “The agro-ecotypes of common bean (*Phaseolus vulgaris*) from Val Belluna (Veneto region),” in *Proceedings of the XLVIII Italian Society of Agricultural Genetics – SIFV-SIGA Joint Meeting*, (Lecce).

[B66] Pritchard J. K. Stephens M. Donnelly P. (2000). Inference of population structure using multilocus genotype data. *Genetics* 155 945–959. 10.1093/genetics/155.2.945 10835412 PMC1461096

[B67] Rakoczy-Trojanowska M. Bolibok-Bragoszewska H. Bolibok H. (2004). Characteristics and a comparison of three classes of microsatellite-based markers and their application in plants. *Cell. Mol. Biol. Lett.* 9 221–238. 15213804

[B68] Ranalli P. Parisi B. (2018). *Bean and String Bean. Cultivation, Choice of Cultivars and Post-harvest (in Italian)*, 1st Edn. Milan: Edagricole – New Business Media.

[B69] Rendón-Anaya M. Montero-Vargas J. M. Saburido-Álvarez S. Vlasova A. Capella-Gutierrez S. Ordaz-Ortiz J. J. (2017). Genomic history of the origin and domestication of common bean unveils its closest sister species. *Genome Biol.* 18:60. 10.1186/s13059-017-1190-6 28356141 PMC5370463

[B70] Rocha-Guzmán N. E. González-Laredo R. F. Ibarra-Pérez F. J. Nava-Berúmen C. A. Gallegos-Infante J. A. (2007). Effect of pressure cooking on the antioxidant activity of extracts from three common bean (*Phaseolus vulgaris* L.) cultivars. *Food Chem.* 100 31–35. 10.1016/j.foodchem.2005.09.005

[B71] Rodiño A. P. Santalla M. De Ron A. M. Singh S. P. (2003). A core collection of common bean from the Iberian Peninsula. *Euphytica* 131 165–175. 10.1023/A:1023973309788

[B72] Rohlf F. (2000). *NTSYS, Numerical Taxonomy and Multivariate Analysis System ver 2.1 Exeter Software.* New York, NY: Applied Biostatistics Inc.

[B73] Schlötterer C. Pemberton J. (1994). “The use of microsatellites for genetic analysis of natural populations,” in *Molecular Ecology and Evolution: Approaches and Applications*, eds Schierwater B. Streit B. Wagner G. P. DeSalle R. (Basel: Birkhauser), 203–214. 10.1007/978-3-0348-7527-1_11 7994107

[B74] Schuelke M. (2000). An economic method for the fluorescent labeling of PCR fragments. *Nat. Biotechnol.* 18 233–234. 10.1038/72708 10657137

[B75] Shimelis E. A. Rakshit S. K. (2005). Proximate composition and physico-chemical properties of improved dry bean (*Phaseolus vulgaris* L.) varieties grown in Ethiopia. *LWT Food Sci. Technol.* 38 331–338. 10.1016/j.lwt.2004.07.002

[B76] Sica P. Galvao A. Scariolo F. Maucieri C. Nicoletto C. Pilon C. (2021). Effects of drought on yield and nutraceutical properties of beans (*Phaseolus* spp.) traditionally cultivated in Veneto, Italy. *Horticulturae* 7 1–17. 10.3390/horticulturae7020017

[B77] Singleton V. L. Orthofer R. Lamuela-Raventós R. M. (1999). Analysis of total phenols and other oxidation substrates and antioxidants by means of folin-ciocalteu reagent. *Methods Enzymol.* 299 152–178. 10.1016/S0076-6879(99)99017-1

[B78] Spagnoletti Zeuli P. L. Baser N. Riluca M. Laghetti G. Logozzo G. Masi P. (2004). “Valorisation and certification of Italian bean agro-ecotypes (*Phaseolus vulgaris*),” in *Proceedings of the Ecotipi Vegetali Italiani: una Preziosa Risorsa di Variabilità Genetica*, (Roma), 19. (in Italian).

[B79] Tohme J. Gonzalez D. O. Beebe S. Duque M. C. (1996). AFLP analysis of gene pools of a wild bean core collection. *Crop Sci.* 36 1375–1384. 10.2135/cropsci1996.0011183X003600050048x

[B80] Veneto Inside (2020). *Venetian Geography.* (in Italian), Available online at: https://www.venetoinside.com/it/scopri-il-veneto/geografia/ (accessed June 6, 2020)

[B81] Venora G. Grillo O. Ravalli C. Cremonini R. (2009). Identification of Italian landraces of bean (*Phaseolus vulgaris* L.) using an image analysis system. *Sci. Hortic.* 121 410–418. 10.1016/j.scienta.2009.03.014

[B82] Vidal-Valverde C. Frias J. Estrella I. Gorospe M. J. Ruiz R. Bacon J. (1994). Effect of processing on some antinutritional factors of lentils. *J. Agric. Food Chem.* 42 2291–2295. 10.1021/jf00046a039

[B83] White J. W. Montes-R C. (1993). The influence of temperature on seed germination in cultivars of common bean. *J. Exp. Bot.* 44 1795–1800. 10.1093/jxb/44.12.1795 12432039

[B84] Yan W. Hunt L. A. (1999). An equation for modelling the temperature response of plants using only the cardinal temperatures. *Ann. Bot.* 84 607–614. 10.1006/anbo.1999.0955

[B85] Yeh F. Yang R. Boyle T. Ye Z. Mao J. (1997). *POPGENE, The User-Friendly Shareware for Population Genetic Analysis.* Alberta: Molecular Biology and Biotechnology Centre, University of Alberta.

[B86] Yu K. Park S. J. Poysa V. Gepts P. (2000). Integration of simple sequence repeat (SSR) markers into a molecular linkage map of common bean (*Phaseolus vulgaris* L.). *J. Hered.* 91 429–434. 10.1093/jhered/91.6.429 11218079

[B87] Zadoks J. C. Chang T. T. Konzak C. F. (1974). A decimal code for the growth stages of cereals. *Weed Res.* 14 415–421. 10.1111/j.1365-3180.1974.tb01084.x

[B88] Zeven A. C. (1997). The introduction of the common bean (*Phaseolus vulgaris* L.) into Western Europe and the phenotypic variation of dry beans collected in the Netherlands in 1946. *Euphytica* 94 319–328. 10.1023/A:1002940220241

[B89] Zhou G. Wang Q. (2018). A new nonlinear method for calculating growing degree days. *Sci. Rep.* 8:10149. 10.1038/s41598-018-28392-z PMC6033920 29977001

