# Supplementary material for: Molecular Hallmarks, Agronomic Performances and Seed Nutraceutical Properties to Exploit Neglected Genetic Resources of Common Beans Grown by Organic Farming in Two Contrasting Environments
Source: Front Plant Sci. 2021 May 25;12:674985. doi: 10.3389/fpls.2021.674985 (PMC8185351; doi:10.3389/fpls.2021.674985)
Supplement: Supplementary file 1 [file Data_Sheet_1.docx]

Supplementary Material

# Supplementary Tables

**Table S1.** List of accessions from DAFNAE’s bean germplasm. The 26 accessions used in this study are highlighted in grey. Accessions that are not highlighted were not assessed in this study due to low germination and/or low availability of seeds.

| Accession Id | Name | | Type | Growth |
| --- | --- | --- | --- | --- |
| 1 | | Fagiolino mangiatutto rampicante | Italian elite lineages | Indeterminate (climbing) |
| 2 | | Borlotto nano | Italian elite lineages | Determined (dwarf) |
| 3 | | Borlotto nano | Italian elite lineages | Determined (dwarf) |
| 4 | | Fagiolo bianco nano | Italian elite lineages | Determined (dwarf) |
| 5 | | Fagiolo rampicante | Italian elite lineages | Indeterminate (climbing) |
| 6 | | Fagiolo nano creso | Italian elite lineages | Determined (dwarf) |
| 7 | | Fagiolo rampicante mangiatutto  (blue lake sel. Gia) | Italian elite lineages | Indeterminate (climbing) |
| 8 | | Fagiolo nano mangiatutto anellino di trento | Italian elite lineages | Determined (dwarf) |
| 9 | | Fagiolo rampicante mangiatutto anellino giallo | Italian elite lineages | Indeterminate (climbing) |
| 10 | | Fagiolo rampicante stortino di trento | Italian elite lineages | Indeterminate (climbing) |
| 11 | | Fagiolo borlotto rampicante  (bortollo lingua di fuoco 3) | Italian elite lineages | Indeterminate (climbing) |
| 12 | | Fagiolo rampicante  (blue lake a grano nero sel. Tom) | Italian elite lineages | Indeterminate (climbing) |
| 13 | | Fagiolo dolico (nano dall'occhio) | Italian elite lineages | Determined (dwarf) |
| 14 | | Fagiolo nano mangiatutto (OR arno) | Italian elite lineages | Determined (dwarf) |
| 15 | | Fagiolo nano (montalbano) | Italian elite lineages | Determined (dwarf) |
| 16 | | Fagiolo rampicante (dolico del metro) | Italian elite lineages | Indeterminate (climbing) |
| 17 | | Fagiolo nano valdarno | Italian elite lineages | Determined (dwarf) |
| 18 | | Fabiolo nano coco nain blanc precoce (lotto verdone) | Italian elite lineages | Determined (dwarf) |
| 19 | | Fagiolo rampicante tondino abruzzese | Italian elite lineages | Indeterminate (climbing) |
| 20 | | Fagiolo verdone del piave  (professional seed) | Italian elite lineages | Determined (dwarf) |
| 21 | | Fagiolo dolico rampicante mangiatutto o stringa | Italian elite lineages | Indeterminate (climbing) |
| 22 | | Fasole del diavolo | Venetians niche populations | Indeterminate (climbing) |
| 23 | | Gialet | Venetians niche populations | Indeterminate (climbing) |
| 24 | | Posenati | Venetians niche populations | Indeterminate (climbing) |
| 25 | | Semi-rampicante abruzzese | Venetians niche populations | Indeterminate (climbing) |
| 26 | | Fasol dela nonna | Venetians niche populations | Indeterminate (climbing) |
| 27 | | Maseleta rossa | Venetians niche populations | Indeterminate (climbing) |
| 28 | | Zia Orsolina | Venetians niche populations | Indeterminate (climbing) |
| 29 | | Meraviglia di Venezia | Venetians niche populations | Indeterminate (climbing) |
| 30 | | Secle | Venetians niche populations | Indeterminate (climbing) |
| 31 | | Della Clorinda | Venetians niche populations | Indeterminate (climbing) |
| 32 | | Pegaso | Venetians niche populations | Indeterminate (climbing) |
| 33 | | SC-iosela | Venetians niche populations | Indeterminate (climbing) |
| 34 | | D'oro (val di fiemme) | Venetians niche populations | Indeterminate (climbing) |
| 35 | | Meso e Meso | Venetians niche populations | Indeterminate (climbing) |
| 36 | | Maron | Venetians niche populations | Indeterminate (climbing) |
| 37 | | Righetti 1 | Venetians niche populations | Indeterminate (climbing) |
| 38 | | Verdine | Venetians niche populations | Indeterminate (climbing) |
| 39 | | Cuna | Venetians niche populations | Indeterminate (climbing) |
| 40 | | Oci Della Madona | Venetians niche populations | Indeterminate (climbing) |
| 41 | | Sciosele | Venetians niche populations | Indeterminate (climbing) |
| 42 | | Monachelle | Venetians niche populations | Indeterminate (climbing) |
| 43 | | Righetti 2 | Venetians niche populations | Indeterminate (climbing) |
| 44 | | Mamme bianche di Bassano - Prod 2018 Azienda | Venetians niche populations | Indeterminate (climbing) |
| 45 | | Mame Bianche B1 - Prod Azienda 2018 | Venetians niche populations | Indeterminate (climbing) |
| 46 | | Banel fonzaso - Prod Azienda 2018 | Venetians niche populations | Indeterminate (climbing) |
| 47 | | Zolferini Rovizetti - Prod Azienda 2018 | Venetians niche populations | Indeterminate (climbing) |
| 48 | | Bala rossa | Venetians niche populations | Indeterminate (climbing) |

**Table S2.** Information on the regions analyzed and SNPs characteristics, including amplicons length and composition (exon length and intron length), SNPs number, typology (synonymous, nonsynonymous, indel), and incidence of SNPs in the different genetic portions. Data are reported for each marker and the total analyzed region.

|  | β-EG | HSF | H4 | β-GBP | PEPC | SHP | LEA | STK | NR | LOX | β-AM | TOT |
| --- | --- | --- | --- | --- | --- | --- | --- | --- | --- | --- | --- | --- |
| Fragment length (bp) | 597 | 720 | 409 | 576 | 529 | 447 | 680 | 571 | 818 | 574 | 612 | 6533 |
| Exon length (bp) | 597 | 299 | 295 | 576 | 420 | 54 | 388 | 188 | 139 | 244 | 428 | 3628 |
| Intron length (bp) | 0 | 421 | 114 | 0 | 109 | 393 | 292 | 383 | 679 | 330 | 184 | 2905 |
| SNP number | 3 | 3 | 7 | 3 | 3 | 10 | 1 | 3 | 2 | 13 | 0 | 48 |
| SNPs in introns | 0 | 2 | 3 | 0 | 0 | 10 | 0 | 2 | 2 | 12 | - | 31 |
| SNPs in exons | 3 | 1 | 4 | 3 | 3 | 0 | 1 | 1 | 0 | 1 | - | 17 |
| Synonymous | 2 | 1 | 0 | 1 | 3 | - | 0 | 1 | - | 1 | - | 9 |
| Nonsynonymous | 1 | 0 | 4 | 2 | 0 | - | 1 | 0 | - | 0 | - | 8 |
| Indels | 0 | 0 | 0 | 0 | 0 | 3 | 0 | 1 | 0 | 4 | 0 | 8 |

**Table S3.** Variable sites in the 10 loci considered, corresponding to 51 SNPs and 4 indels. Haplotypes (Haplo01-Haplo21) are ordered based on the UPGMA clustering, and the number of entries for each haplogroup is reported. The consensus sequence and the alternative nucleotide are reported for each position. Mutations that caused an amino acid substitution are marked with an asterisk on the consensus sequence.

| d |  | Entries | β-EG | | | HSF | | | LEA | STK | | | | | | | | NR | | LOX | | | | | | | | | | | | | | | | | | | |
| --- | --- | --- | --- | --- | --- | --- | --- | --- | --- | --- | --- | --- | --- | --- | --- | --- | --- | --- | --- | --- | --- | --- | --- | --- | --- | --- | --- | --- | --- | --- | --- | --- | --- | --- | --- | --- | --- | --- | --- |
| Cluster A | Hap01 | 45 |  |  |  | T | T | C |  |  |  |  |  |  |  |  |  |  |  |  |  |  |  |  |  |  |  |  |  |  |  |  |  |  |  |  |  |  | |
|  | Hap02 | 21 |  |  |  |  |  |  |  |  |  |  |  |  |  |  |  |  |  |  |  |  |  |  |  |  |  |  |  |  |  |  |  |  |  |  |  |  | |
|  | Hap03 | 5 |  |  |  | T | T | C |  |  |  |  |  |  |  |  |  |  |  |  |  |  |  |  |  |  |  |  |  |  |  |  |  |  |  |  |  |  | |
|  | Hap04 | 5 |  |  |  | T | T | C |  | _ | _ | _ | _ | _ |  |  |  |  |  |  |  |  |  |  |  |  |  |  |  |  |  |  |  |  |  |  |  |  | |
|  | Hap05 | 4 |  |  |  |  |  |  |  |  |  |  |  |  |  |  |  |  |  |  |  |  |  |  |  |  |  |  |  |  |  |  |  |  |  |  |  |  | |
|  | Hap06 | 3 |  |  |  | T | T | C |  |  |  |  |  |  |  |  |  |  |  |  |  |  |  |  |  |  |  |  |  |  |  |  |  |  |  |  |  |  | |
|  | Hap07 | 1 |  |  |  | T | T | C |  |  |  |  |  |  |  |  |  |  |  |  |  |  |  |  |  |  | T | C | _ | _ | _ | _ | C |  | C |  |  |  | |
| Cluster B | Hap08 | 18 |  |  |  | T | T | C |  |  |  |  |  |  |  |  |  |  |  |  |  |  |  |  |  |  |  |  |  |  |  |  |  |  |  |  |  |  | |
|  | Hap09 | 13 | C | G | C |  |  |  |  |  |  |  |  |  |  |  |  | G |  | _ | _ | _ | C | T | C | T | T |  |  |  |  |  |  |  | C |  | A |  | |
|  | Hap10 | 12 | C | G | C |  |  |  | G |  |  |  |  |  |  |  | T | G |  |  |  |  |  |  |  |  | T | C | _ | _ | _ | _ | C |  | C | C |  | G | |
|  | Hap11 | 12 | C | G | C |  |  |  |  | _ | _ | _ | _ | _ | C | T |  |  |  |  |  |  |  |  |  |  | T | C | _ | _ | _ | _ | C |  | C |  |  |  | |
|  | Hap12 | 8 |  |  |  |  |  |  |  |  |  |  |  |  |  |  |  |  |  | _ | _ | _ | C | T | C | T | T |  |  |  |  |  |  |  | C |  | A |  | |
|  | Hap13 | 5 | C | G | C |  |  |  | G |  |  |  |  |  |  |  |  | G | A |  | T |  |  | T |  |  | T |  |  |  |  |  |  | G | C |  |  | G | |
|  | Hap14 | 4 |  |  |  |  |  |  |  |  |  |  |  |  |  |  |  | G | A |  | T |  |  | T |  |  | T |  |  |  |  |  |  | G | C |  |  | G | |
|  | Hap15 | 4 | C | G | C |  |  |  |  |  |  |  |  |  |  |  |  | G |  |  | T |  |  | T |  |  | T |  |  |  |  |  |  | G | C |  |  | G | |
|  | Hap16 | 3 |  |  |  |  |  |  |  |  |  |  |  |  |  |  |  |  |  |  |  |  |  |  |  |  |  |  |  |  |  |  |  |  |  |  |  |  | |
|  | Hap17 | 2 | C | G | C |  |  |  |  |  |  |  |  |  |  |  |  |  |  | _ | _ | _ | C | T | C | T | T |  |  |  |  |  |  |  | C |  | A |  | |
|  | Hap18 | 1 | C | G | C |  |  |  |  |  |  |  |  |  |  |  |  | G |  | _ | _ | _ | C | T | C | T | T |  |  |  |  |  |  |  | C |  | A |  | |
|  | Hap19 | 1 | C | G | C | T | T | C |  |  |  |  |  |  |  |  |  | G |  | _ | _ | _ | C | T | C | T | T |  |  |  |  |  |  |  | C |  | A |  | |
|  | Hap20 | 1 | C | G | C |  |  |  |  |  |  |  |  |  |  |  |  | G |  | _ | _ | _ | C | T | C | T | T |  |  |  |  |  |  |  | C |  | A |  | |
|  | Hap21 | 1 |  |  |  | T | T | C |  |  |  |  |  |  |  |  |  | G |  |  | T |  |  | T |  |  | T |  |  |  |  |  |  | G | C |  |  | G | |
|  | CONSENSUS |  | A | A | G* | G | C | T | T* | C | A | A | A | C | T | C | G | T | C | C | A | T | T | G | _ | C | C | T | T | A | A | C | T | A | A | T | G | T | |
|  |  | Entries | H4 | | | | | | | β-GBP | | | PEPC | | | SHP | | | | | | | | | | | | | | | | | | | | | | |  |
| Cluster A | Hap01 | 45 |  |  |  |  |  |  |  |  |  |  |  |  |  |  |  |  |  |  |  |  |  |  |  |  |  |  |  |  |  |  |  |  |  |  |  |  | |
|  | Hap02 | 21 |  |  |  |  |  |  |  |  |  |  |  |  |  |  |  |  |  |  |  |  |  |  |  |  |  |  |  |  |  |  |  |  |  |  |  |  | |
|  | Hap03 | 5 |  |  |  |  |  |  |  |  |  |  |  |  |  |  |  |  |  |  |  |  |  |  |  |  |  |  |  |  |  |  |  |  |  |  |  |  | |
|  | Hap04 | 5 |  |  |  |  |  |  |  |  |  |  |  |  |  |  |  |  |  |  |  |  |  |  |  |  |  |  |  |  |  |  |  |  |  |  |  |  | |
|  | Hap05 | 4 |  |  |  |  |  |  |  |  |  |  | T |  | G |  |  |  |  |  |  |  |  |  |  |  |  |  |  |  |  |  |  |  |  |  |  |  | |
|  | Hap06 | 3 |  |  |  |  |  |  |  |  |  | A |  |  |  |  |  |  |  |  |  |  |  |  |  |  |  |  |  |  |  |  |  |  |  |  |  |  | |
|  | Hap07 | 1 |  |  |  |  |  |  |  | A | A |  |  |  |  |  |  |  |  |  |  |  |  |  |  |  |  |  |  |  |  |  |  |  |  |  |  |  | |
| Cluster B | Haplo_08 | 18 |  |  |  |  |  |  |  |  |  |  |  |  |  | T | A | T | _ | G | _ | _ | _ | _ | G | C | C | A | T | G | T | C | A | T | T | A | C |  | |
|  | Haplo_09 | 13 | G | A | A | A | A | G | A | A | A |  |  | A |  | T | A | T | _ | G | _ | _ | _ | _ | G | C | C | A | T | G | T | C | A | T | T | A | C |  | |
|  | Haplo_10 | 12 | G | A | A |  | A | G | A | A | A |  | T |  | G | T | A | T | _ | G | _ | _ | _ | _ | G | C | C | A | T | G | T | C | A | T | T | A | C |  | |
|  | Haplo_11 | 12 | G | A | A |  | A | G | A | A | A |  | T |  | G | T | A | T | _ | G | _ | _ | _ | _ | G | C | C | A | T | G | T | C | A | T | T | A | C |  | |
|  | Haplo_12 | 8 |  |  |  |  |  |  |  | A | A |  |  | A |  | T | A | T | _ | G | _ | _ | _ | _ | G | C | C | A | T | G | T | C | A | T | T | A | C |  | |
|  | Haplo_13 | 5 | G | A | A |  | A | G | A | A | A |  | T |  | G | T | A | T | _ | G | _ | _ | _ | _ | G | C | C | A | T | G | T | C | A | T | T | A | C |  | |
|  | Haplo_14 | 4 | G | A | A |  | A | G | A | A | A |  |  | A |  | T | A | T | _ | G | _ | _ | _ | _ | G | C | C | A | T | G | T | C | A | T | T | A | C |  | |
|  | Haplo_15 | 4 | G | A | A |  | A | G | A | A | A |  | T |  | G | T | A | T | _ | G | _ | _ | _ | _ | G | C | C | A | T | G | T | C | A | T | T | A | C |  | |
|  | Haplo_16 | 3 |  |  |  |  |  |  |  |  |  |  |  |  |  | T | A | T | _ | G | _ | _ | _ | _ | G | C | C | A | T | G | T | C | A | T | T | A | C |  | |
|  | Haplo_17 | 2 | G | A | A | A | A | G | A | A | A |  |  | A |  | T | A | T | _ | G | _ | _ | _ | _ | G | C | C | A | T | G | T | C | A | T | T | A | C |  | |
|  | Haplo_18 | 1 |  |  |  |  |  |  |  |  |  |  |  | A |  | T | A | T | _ | G | _ | _ | _ | _ | G | C | C | A | T | G | T | C | A | T | T | A | C |  | |
|  | Haplo_19 | 1 |  |  |  |  |  |  |  |  |  |  |  | A |  | T | A | T | _ | G | _ | _ | _ | _ | G | C | C | A | T | G | T | C | A | T | T | A | C |  | |
|  | Haplo_20 | 1 |  |  |  |  | A | G |  |  |  |  |  | A |  | T | A | T | _ | G | _ | _ | _ | _ | G | C | C | A | T | G | T | C | A | T | T | A | C |  | |
|  | Haplo_21 | 1 | G | A | A |  | A | G | A |  |  |  |  | A |  | T | A | T | _ | G | _ | _ | _ | _ | G | C | C | A | T | G | T | C | A | T | T | A | C |  | |
|  | CONSENSUS |  | T* | G* | G* | G* | G | A | G | T* | G | C* | C | T | A | C | G | C | T | T | G | A | T | A | T | G | A | T | C | _ | _ | _ | _ | _ | _ | _ | T |  | |

**Table S4.** Relative frequency (%) of the SNP-derived allele variants found in the core collection for each of the 11 target genes, number of polymorphic sites (S) at each locus, relative number of identified alleles (Hp n°), Nei’s haplotype distance (Hp d.) (Nei, 1987). Information are calculated for each of the four identified clusters, based on variety identity (Italian elite lineages and Venetians niche populations) or ancestry identity (Andean and Mesoamerican), and in total

| **Total** | β-EG | HSF | H4 | β-GBP | PEPC | SHP | LEA | STK | NR | LOX | β-AM |
| --- | --- | --- | --- | --- | --- | --- | --- | --- | --- | --- | --- |
| Allele A | 75.60 | 53.70 | 75.60 | 73.20 | 61.00 | 53.70 | 90.20 | 90.30 | 75.60 | 61.00 | 100.00 |
| Allele B | 24.40 | 41.50 | 14.60 | 24.40 | 22.00 | 46.30 | 4.90 | 4.90 | 19.50 | 14.60 |  |
| Allele C |  |  | 7.30 | 2.40 | 14.60 |  |  | 2.40 | 4.90 | 9.80 |  |
| Allele D |  |  | 2.40 |  |  |  |  | 2.40 |  | 7.30 |  |
| Allele E |  |  |  |  |  |  |  |  |  | 2.40 |  |
| S | 3 | 3 | 7 | 3 | 3 | 22 | 1 | 8 | 2 | 19 | 0 |
| Hp n° | 2 | 2 | 4 | 3 | 3 | 2 | 2 | 4 | 3 | 5 | 1 |
| Hp d. | 0.68 | 0.77 | 0.85 | 0.80 | 0.85 | 0.75 | 0.59 | 0.80 | 0.80 | 0.92 | 0.00 |
| **Italian elite lineages** | β-EG | HSF | H4 | β-GBP | PEPC | SHP | LEA | STK | NR | LOX | β-AM |
| Allele A | 70.00 | 35.00 | 70.00 | 65.00 | 55.00 | 50.00 | 100.00 | 90.00 | 65.00 | 50.00 | 100.00 |
| Allele B | 30.00 | 65.00 | 10.00 | 30.00 | 45.00 | 50.00 |  | 10.00 | 30.00 | 30.00 |  |
| Allele C |  |  | 15.00 | 5.00 |  |  |  |  | 5.00 | 10.00 |  |
| Allele D |  |  | 5.00 |  |  |  |  |  |  | 10.00 |  |
| Allele E |  |  |  |  |  |  |  |  |  |  |  |
| Hp n° | 2 | 2 | 4 | 3 | 2 | 2 | 1 | 2 | 3 | 4 | 1 |
| Hp d. | 0.71 | 0.73 | 0.87 | 0.83 | 0.75 | 0.75 | 0.00 | 0.59 | 0.83 | 0.91 | 0.00 |
| **Venetians niche populations** | β-EG | HSF | H4 | β-GBP | PEPC | SHP | LEA | STK | NR | LOX | β-AM |
| Allele A | 80.95 | 76.19 | 80.95 | 80.95 | 66.67 | 57.14 | 80.95 | 90.48 | 85.71 | 71.43 | 100.00 |
| Allele B | 19.05 | 23.81 | 19.05 | 19.05 |  | 42.86 | 9.52 |  | 9.52 |  |  |
| Allele C |  |  |  |  | 23.81 |  |  | 4.76 | 4.76 | 9.52 |  |
| Allele D |  |  |  |  |  |  |  | 4.76 |  | 4.76 |  |
| Allele E |  |  |  |  |  |  |  |  |  | 4.76 |  |
| Hp n° | 2 | 2 | 2 | 2 | 2 | 2 | 2 | 3 | 3 | 4 | 1 |
| Hp d. | 0.65 | 0.68 | 0.65 | 0.65 | 0.75 | 0.74 | 0.67 | 0.73 | 0.75 | 0.87 | 0.00 |
| **Andean** | β-EG | HSF | H4 | β-GBP | PEPC | SHP | LEA | STK | NR | LOX | β-AM |
| Allele A | 100.00 | 40.91 | 100.00 | 90.91 | 90.91 | 100.00 | 90.91 | 90.91 | 100.00 | 86.36 | 100.00 |
| Allele B |  | 59.09 |  | 4.55 |  |  |  | 9.09 |  |  |  |
| Allele C |  |  |  | 4.55 | 9.09 |  |  |  |  |  |  |
| Allele D |  |  |  |  |  |  |  |  |  | 4.55 |  |
| Allele E |  |  |  |  |  |  |  |  |  |  |  |
| Hp n° | 1 | 2 | 1 | 3 | 2 | 1 | 1 | 2 | 1 | 2 | 1 |
| Hp d. | 0.00 | 0.74 | 0.00 | 0.72 | 0.58 | 0.00 | 0.17 | 0.58 | 0.00 | 0.63 | 0.00 |
| **Mesoamerican** | β-EG | HSF | H4 | β-GBP | PEPC | SHP | LEA | STK | NR | LOX | β-AM |
| Allele A | 47.37 | 73.68 | 42.11 | 52.63 | 26.32 |  | 89.47 | 89.47 | 47.37 | 31.58 | 100.00 |
| Allele B | 52.63 | 26.32 | 31.58 | 47.37 | 47.37 | 100.00 | 10.53 |  | 42.11 | 31.58 |  |
| Allele C |  |  | 15.79 |  | 21.05 |  |  | 5.26 | 10.53 | 21.05 |  |
| Allele D |  |  | 5.26 |  |  |  |  | 5.26 |  | 10.53 |  |
| Allele E |  |  |  |  |  |  |  |  |  | 5.26 |  |
| Hp n° | 2 | 2 | 4 | 2 | 3 | 1 | 2 | 3 | 3 | 5 | 1 |
| Hp d. | 0.75 | 0.69 | 0.92 | 0.75 | 0.89 | 0.00 | 0.59 | 0.73 | 0.86 | 0.95 | 0.00 |

**Table S5.** Effects of environments and accession on seeds’ physical characteristics and yield of dwarf accessions.

|  | Weight 100 seeds (g) | Volume of 10 seeds (mL) | Seed density (g mL^-1^) | Length (mm) | Width (mm) | Thickness (mm) | Yield  (g per plant) |
| --- | --- | --- | --- | --- | --- | --- | --- |
|  |  |  |  |  |  |  |  |
| *Environment* | | | | | | | |
| Mountain | 44.8 A | 3.6 A | 1.26 A | 13.4 A | 7.6 A | 6.4 A | 54.1 n.s. |
| Sea level | 37.7 B | 3.2 B | 1.14 B | 12.4 B | 7.0 B | 5.8 B | 56.8 n.s. |
| *Accession* | | | | | | | |
| 2 | 60.3 a | 5.4 a | 1.11 n.s. | 14.5 ab | 9.5 a | 7.0 ab | 53.8 abc |
| 3 | 61.7 a | 5.3 a | 1.15 n.s. | 15.3 a | 9.0 a | 7.7 a | 42.1 abc |
| 6 | 30.2 d | 2.5 c | 1.21 n.s. | 12.5 c | 5.9 cd | 5.5 cd | 82.7 a |
| 8 | 36.2 c | 2.9 c | 1.24 n.s. | 13.1 bc | 6.4 c | 5.8 c | 59.1 abc |
| 17 | 19.2 e | 1.6 d | 1.22 n.s. | 10.2 d | 5.2 d | 4.8 d | 27.7 bc |
| 18 | 35.3 c | 2.8 c | 1.29 n.s. | 10.4 d | 7.5 b | 6.3 bc | 67.4 ab |
| 20 | 46.0 b | 3.9 b | 1.78 n.s. | 14.1 ab | 7.5 b | 5.9 c | 20.3 c |

Different uppercase letters in the same column indicate a significant difference between environments. Different lowercase letters in the same column indicate a significant difference between accessions. n.s. = non-significant genotypic effect. α = 0.05.

**Table S6.** Effects of the interaction between accession and environment on climbing accessions seeds’ physical characteristics. Different letters in the same column indicate a significant difference between compared groups. α = 0.05.

|  |  | Weight of  100 seeds (g) | Volume  of 10 seeds (mL) | Seed  density  (g mL^-1^) | Length (mm) | Width (mm) | Thickness (mm) |
| --- | --- | --- | --- | --- | --- | --- | --- |
| Mountain | 1 | 83 abcdef | 6.8 abcd | 1.22 abcd | 17.3 abc | 8.9 ghijkl | 7.0 bcdefghijklmn |
|  | 7 | 27 rs | 2.0 m | 1.25 abcd | 13.0 fghijklm | 6.4 no | 5.5 klmnopq |
|  | 9 | 68 ghij | 5.3 ef | 1.29 abcd | 16.5 abcde | 9.5 defghijk | 6.9 cdefghijklmn |
|  | 11 | 77 bcdefghi | 6.8 abcd | 1.12 bcd | 15.1 abcdefghij | 10.9 abcde | 6.8 defghijklmno |
|  | 12 | 30 qrs | 2.5 klm | 1.21 abcd | 13.2 fghijklm | 5.8 o | 4.7 q |
|  | 19 | 44 mnopq | 3.3 jkl | 1.33 abc | 10.5 m | 8.9 ghijkl | 7.0 cdefghijklmn |
|  | 23 | 47 mnop | 3.5 ijk | 1.33 abc | 12.2 ijklm | 8.4 hijklm | 6.5 fghijklmnopq |
|  | 24 | 90 ab | 7.2 ab | 1.26 abcd | 15.8 abcdefg | 11.0 abcd | 8.6 abcd |
|  | 25 | 40 mnopqr | 3.0 jklm | 1.32 abc | 12.5 hijklm | 9.0 efghijk | 5.3 mnopq |
|  | 26 | 46 mnop | 3.3 jkl | 1.39 a | 11.3 klm | 8.7 hijkl | 6.7 efghijklmnop |
|  | 27 | 69 fghi | 5.2 efg | 1.34 ab | 12.1 jklm | 9.8 cdefghi | 7.9 abcdefgh |
|  | 28 | 90 ab | 7.0 abc | 1.28 abcd | 15.4 abcdefghi | 10.8 abcdef | 8.4 abcde |
|  | 29 | 39 nopqrs | 3.2 jklm | 1.23 abcd | 14.1 cdefghijkl | 7.8 klmn | 4.8 pq |
|  | 30 | 71 defghi | 5.8 cdef | 1.21 abcd | 14.5 abcdefghijk | 9.8 cdefghij | 7.6 abcdefghij |
|  | 31 | 64 hijk | 5.0 efg | 1.28 abcd | 16.1 abcdef | 10.2 bcdefgh | 6.5 efghijklmnopq |
|  | 32 | 82 abcdefg | 6.8 abcd | 1.20 abcd | 17.6 a | 9.6 defghijk | 6.1 hijklmnopq |
|  | 33 | 94 a | 7.5 a | 1.25 abcd | 15.8 abcdefgh | 11.8 ab | 8.4 abcdef |
|  | 34 | 85 abcde | 6.2 bcde | 1.37 ab | 14.1 cdefghijkl | 10.2 bcdefgh | 8.9 ab |
|  | 36 | 91 ab | 7.3 ab | 1.25 abcd | 17.0 abcd | 12.2 a | 9.4 a |
| 1 | 79 bcdefgh | 7.0 abc | 1.13 bcd | 14.9 abcdefghij | 8.0 jklmn | 5.5 lmnopq |  |
| Sea level | 7 | 25 s | 2.2 lm | 1.23 abcd | 12.4 hijklm | 6.7 mno | 5.2 nopq |
|  | 9 | 46 mnop | 4.0 ghij | 1.15 abcd | 14.1 cdefghijkl | 8.0 ijklmn | 5.8 jklmnopq |
|  | 11 | 81 abcdefg | 7.5 a | 1.08 cd | 17.4 ab | 11.0 abcd | 8.8 abc |
|  | 12 | 37 opqrs | 3.2 jklm | 1.15 abcd | 13.7 efghijklm | 7.0 klmno | 5.0 opq |
|  | 19 | 45 mnop | 3.7 hijk | 1.24 abcd | 11.5 klm | 9.5 defghijk | 7.8 abcdefghi |
|  | 23 | 44 mnopq | 3.5 ijk | 1.25 abcd | 12.6 ghijklm | 7.9 klmn | 6.3 ghijklmnopq |
|  | 24 | 74 cdefghi | 6.2 bcde | 1.21 abcd | 15.4 abcdefgh | 10.3 abcdefgh | 7.4 bcdefghijkl |
|  | 25 | 39 nopqrs | 3.3 jkl | 1.17 abcd | 11.9 jklm | 7.7 klmn | 5.6 klmnopq |
|  | 26 | 49 lmno | 4.0 ghij | 1.23 abcd | 11.0 lm | 9.6 defghijk | 7.4 bcdefghijk |
|  | 27 | 63 ijkl | 5.2 efg | 1.22 abcd | 13.2 fghijklm | 10.2 bcdefgh | 7.8 abcdefghi |
|  | 28 | 85 abcd | 7.2 ab | 1.18 abcd | 14.2 cdefghijklm | 10.7 abcdefg | 8.0 abcdefg |
|  | 29 | 34 pqrs | 3.2 jklm | 1.07 d | 13.1 fghijklm | 7.8 abcdefg | 5.5 lmnopq |
|  | 30 | 54 jklm | 4.7 fghi | 1.16 abcd | 13.3 efghijklm | 8.9 fghijk | 6.9 defghijklm |
|  | 31 | 54 jklm | 4.8 fgh | 1.12 bcd | 13.2 fghijklm | 8.4 hijklm | 6.0 ijklmnopq |
|  | 32 | 87 abc | 7.7 a | 1.14 abcd | 17.3 abc | 9.8 cdefghij | 7.1 bcdefghijklm |
|  | 33 | 73 cdefghi | 6.2 bcde | 1.19 abcd | 15.8 abcdefg | 11.6 abc | 7.3 bcdefghijkl |
|  | 34 | 53 klmn | 4.0 ghij | 1.31 abcd | 13.8 defghijklm | 8.8 ghijkl | 8.1 abcdefg |
|  | 36 | 70 efghi | 5.7 def | 1.24 abcd | 13.8 defghijklm | 9.0 efghijk | 7.1 bcdefghijklm |

**Table S7.** Effects of the accession on the essential amino acids profile of climbing and dwarf accesions. Different letters indicate a significant difference between accessions. n.s. = non-significant effect. α = 0.05.

|  | Histidine^ns^ | Methionine^*^ | Phenylalanine^ns^ | Isoleucine^ns^ | Leucine^ns^ | Lysine^ns^ | Threonine^*^ | Tryptophan^ns^ | Valine^ns^ |
| --- | --- | --- | --- | --- | --- | --- | --- | --- | --- |
| Climbing (mg 100 g^-1^) | | | | | | | | | |
| 1 | 675 | 321a | 1492 | 1053 | 1985 | 2612 | 1261a | 175 | 1161 |
| 7 | 762 | 253c | 1380 | 968 | 1875 | 2499 | 1197ab | 188 | 1048 |
| 9 | 839 | 292abc | 1455 | 1027 | 2021 | 2938 | 1178ab | 169 | 1116 |
| 11 | 497 | 273abc | 1202 | 836 | 1658 | 1816 | 1091ab | 174 | 947 |
| 12 | 531 | 256c | 1234 | 850 | 1671 | 1939 | 1081ab | 165 | 949 |
| 19 | 467 | 256c | 1133 | 806 | 1531 | 1750 | 970ab | 184 | 906 |
| 23 | 678 | 263bc | 1264 | 937 | 1841 | 2233 | 1026ab | 198 | 1031 |
| 24 | 803 | 293abc | 1402 | 1022 | 1986 | 2795 | 1181ab | 195 | 1136 |
| 25 | 524 | 261bc | 1099 | 817 | 1595 | 1760 | 899b | 174 | 893 |
| 26 | 445 | 256c | 1068 | 775 | 1470 | 1657 | 958ab | 210 | 881 |
| 27 | 521 | 282abc | 1227 | 868 | 1694 | 1888 | 962ab | 175 | 970 |
| 28 | 495 | 277abc | 1207 | 868 | 1648 | 1787 | 990ab | 177 | 973 |
| 29 | 511 | 260bc | 1348 | 1003 | 1932 | 2111 | 1042ab | 199 | 1100 |
| 30 | 436 | 267abc | 1174 | 847 | 1619 | 1728 | 911b | 172 | 960 |
| 31 | 529 | 280abc | 1244 | 885 | 1705 | 1959 | 945ab | 180 | 1009 |
| 32 | 659 | 283abc | 1521 | 1084 | 2086 | 2621 | 1093ab | 185 | 1214 |
| 33 | 631 | 277abc | 1371 | 981 | 1891 | 2403 | 1015ab | 194 | 1084 |
| 34 | 612 | 284abc | 1378 | 991 | 1871 | 2295 | 1027ab | 178 | 1094 |
| 36 | 425 | 310ab | 1263 | 907 | 1730 | 1821 | 1000ab | 171 | 1042 |
|  | Histidine^ns^ | Methionine^n.s^ | Phenylalanine^ns^ | Isoleucine^ns^ | Leucine^ns^ | Lysine^ns^ | Threonine^*^ | Tryptophan^ns^ | Valine^ns^ |
| Dwarf (mg 100 g^-1^) | | | | | | | | | |
| 2 | 491 | 272 | 1332 | 913 | 1796 | 2032 | 1179a | 169 | 1052 |
| 3 | 546 | 279 | 1199 | 817 | 1569 | 1774 | 1003c | 156 | 917 |
| 6 | 551 | 239 | 1155 | 793 | 1535 | 1807 | 1013c | 169 | 901 |
| 8 | 617 | 294 | 1310 | 906 | 1784 | 2122 | 1131ab | 161 | 1027 |
| 17 | 650 | 224 | 1269 | 899 | 1723 | 2235 | 1023c | 156 | 996 |
| 18 | 496 | 236 | 1185 | 840 | 1629 | 1771 | 1045c | 191 | 934 |
| 20 | 542 | 276 | 1327 | 930 | 1796 | 2075 | 1100b | 179 | 1059 |

**Table S8.** Effects of the accessions on the non-essential amino acids profile of climbing and dwarf accessions.

|  | Aspartic acid^ns^ | Glutamic acid^ns^ | Serine^ns^ | Glycine^ns^ | Arginine^ns^ | Alanine^ns^ | Tyrosine^*^ | Proline^ns^ | Cysteine^ns^ |
| --- | --- | --- | --- | --- | --- | --- | --- | --- | --- |
| Climbing (mg 100 g^-1^) | | | | | | | | | |
| 1 | 3396 | 5113 | 1742 | 1064 | 1778 | 1146 | 811a | 1372 | 289 |
| 7 | 3006 | 4714 | 1539 | 992 | 1437 | 1073 | 726ab | 1209 | 216 |
| 9 | 3326 | 5253 | 1660 | 1031 | 1774 | 1131 | 778ab | 1326 | 260 |
| 11 | 3075 | 4730 | 1529 | 930 | 1468 | 1044 | 614ab | 1425 | 273 |
| 12 | 2936 | 4322 | 1453 | 928 | 1355 | 1014 | 629ab | 1347 | 238 |
| 19 | 2653 | 4062 | 1314 | 863 | 1227 | 942 | 572ab | 1288 | 226 |
| 23 | 3053 | 4811 | 1507 | 919 | 1564 | 1037 | 658ab | 1938 | 229 |
| 24 | 3361 | 5187 | 1651 | 993 | 1480 | 1134 | 734ab | 1915 | 229 |
| 25 | 2811 | 4585 | 1359 | 863 | 1615 | 969 | 616ab | 1581 | 214 |
| 26 | 2617 | 3829 | 1306 | 837 | 1189 | 934 | 569b | 1461 | 222 |
| 27 | 2961 | 4592 | 1475 | 913 | 1616 | 1013 | 644ab | 1658 | 227 |
| 28 | 2961 | 4631 | 1457 | 889 | 1516 | 1009 | 615ab | 1621 | 242 |
| 29 | 3293 | 5189 | 1644 | 1032 | 1710 | 1133 | 706ab | 1666 | 198 |
| 30 | 2943 | 4245 | 1388 | 890 | 1553 | 981 | 589ab | 1551 | 222 |
| 31 | 2966 | 4501 | 1422 | 935 | 1487 | 1025 | 616ab | 1564 | 206 |
| 32 | 3497 | 5466 | 1738 | 1071 | 1966 | 1180 | 785ab | 1702 | 221 |
| 33 | 3130 | 4839 | 1523 | 970 | 1682 | 1081 | 682ab | 1624 | 213 |
| 34 | 3130 | 4919 | 1545 | 950 | 1406 | 1067 | 704ab | 1598 | 236 |
| 36 | 3181 | 4883 | 1543 | 956 | 1543 | 1074 | 686ab | 1667 | 212 |
|  | Aspartic acid^ns^ | Glutamic acid^ns^ | Serin^*^ | Glycine^*^ | Arginine^ns^ | Alanine^ns^ | Tyrosine^n.s.^ | Proline^ns^ | Cysteine^ns^ |
| Dwarf (mg 100 g^-1^) | | | | | | | | | |
| 2 | 3191 | 4683 | 1611a | 1004a | 1520 | 1104 | 679 | 1415 | 223 |
| 3 | 2733 | 4047 | 1363ab | 852c | 1277 | 941 | 658 | 1314 | 210 |
| 6 | 2656 | 3966 | 1334b | 863bc | 1218 | 943 | 585 | 1125 | 225 |
| 8 | 3135 | 4526 | 1549a | 973abc | 1495 | 1062 | 684 | 1381 | 227 |
| 17 | 2913 | 4388 | 1433a | 916abc | 1435 | 996 | 609 | 1559 | 188 |
| 18 | 2832 | 4170 | 1406ab | 898abc | 1260 | 994 | 590 | 1410 | 215 |
| 20 | 3218 | 4778 | 1567a | 986ab | 1483 | 1097 | 673 | 1485 | 257 |

Different letters indicate a significant difference between accessions n.s. = non-significant effect. α = 0.05.

# Supplementary Figures


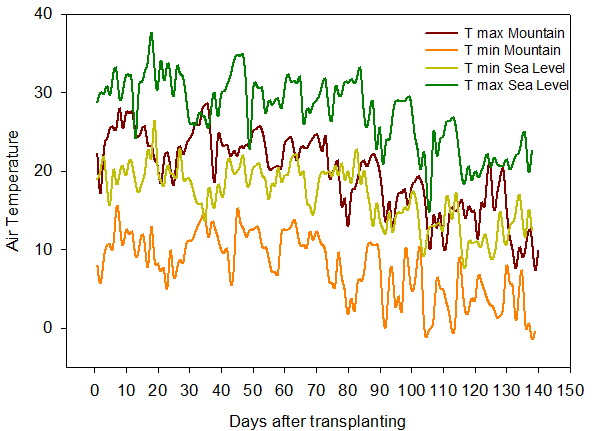


**Figure S1**. Average daily maximum and minimum temperatures in Asiago (mountain) and Legnaro (sea level) during the bean production cycle.

| 1 | 100% |  |  |  |  |  |  |  |  |  |  |  |  |  |  |  |  |  |  |  |  |  |  |  |  |
| --- | --- | --- | --- | --- | --- | --- | --- | --- | --- | --- | --- | --- | --- | --- | --- | --- | --- | --- | --- | --- | --- | --- | --- | --- | --- |
| 2 | 81.3% | 97.1% |  |  |  |  |  |  |  |  |  |  |  |  |  |  |  |  |  |  |  |  |  |  |  |
| 3 | 86.0% | 94.2% | 100% |  |  |  |  |  |  |  |  |  |  |  |  |  |  |  |  |  |  |  |  |  |  |
| 6 | 89.5% | 88.3% | 93.0% | 100% |  |  |  |  |  |  |  |  |  |  |  |  |  |  |  |  |  |  |  |  |  |
| 7 | 68.4% | 67.3% | 68.4% | 71.9% | 100% |  |  |  |  |  |  |  |  |  |  |  |  |  |  |  |  |  |  |  |  |
| 8 | 82.5% | 83.6% | 89.5% | 82.5% | 68.4% | 100% |  |  |  |  |  |  |  |  |  |  |  |  |  |  |  |  |  |  |  |
| 9 | 86.0% | 70.8% | 75.4% | 78.9% | 64.9% | 82.5% | 100% |  |  |  |  |  |  |  |  |  |  |  |  |  |  |  |  |  |  |
| 11 | 89.5% | 75.4% | 78.9% | 78.9% | 68.4% | 78.9% | 78.9% | 100% |  |  |  |  |  |  |  |  |  |  |  |  |  |  |  |  |  |
| 12 | 75.1% | 75.0% | 79.2% | 76.9% | 83.3% | 79.2% | 72.2% | 74.6% | 90.9% |  |  |  |  |  |  |  |  |  |  |  |  |  |  |  |  |
| 17 | 86.0% | 81.3% | 86.0% | 86.0% | 70.2% | 82.5% | 75.4% | 82.5% | 76.0% | 96.5% |  |  |  |  |  |  |  |  |  |  |  |  |  |  |  |
| 18 | 76.4% | 77.3% | 80.0% | 80.0% | 69.1% | 72.8% | 69.1% | 70.9% | 68.2% | 81.0% | 97.5% |  |  |  |  |  |  |  |  |  |  |  |  |  |  |
| 20 | 81.1% | 83.8% | 87.3% | 83.1% | 68.4% | 81.1% | 72.7% | 76.9% | 71.8% | 85.3% | 80.2% | 98.5% |  |  |  |  |  |  |  |  |  |  |  |  |  |
| 23 | 66.0% | 64.0% | 66.0% | 66.0% | 72.0% | 66.0% | 70.0% | 66.0% | 74.0% | 70.0% | 76.1% | 71.6% | 97.3% |  |  |  |  |  |  |  |  |  |  |  |  |
| 24 | 78.9% | 70.8% | 75.4% | 78.9% | 68.4% | 75.4% | 80.7% | 71.9% | 72.8% | 77.2% | 72.4% | 78.5% | 74.0% | 97.7% |  |  |  |  |  |  |  |  |  |  |  |
| 25 | 64.9% | 63.7% | 64.9% | 64.9% | 68.4% | 64.9% | 68.4% | 64.9% | 69.9% | 64.9% | 72.6% | 64.2% | 78.0% | 71.9% | 100% |  |  |  |  |  |  |  |  |  |  |
| 26 | 82.5% | 75.4% | 78.9% | 82.5% | 68.4% | 78.9% | 82.5% | 78.9% | 71.1% | 75.4% | 74.5% | 78.9% | 66.0% | 78.9% | 64.9% | 100% |  |  |  |  |  |  |  |  |  |
| 27 | 78.3% | 73.9% | 73.9% | 78.3% | 69.6% | 78.3% | 82.6% | 73.9% | 69.2% | 76.1% | 71.7% | 79.4% | 69.6% | 89.1% | 65.2% | 87.0% | 100% |  |  |  |  |  |  |  |  |
| 28 | 86.0% | 74.3% | 78.9% | 82.5% | 68.4% | 78.9% | 86.0% | 82.5% | 71.6% | 80.7% | 74.6% | 81.1% | 70.0% | 89.5% | 64.9% | 86.0% | 91.3% | 100% |  |  |  |  |  |  |  |
| 29 | 64.9% | 67.3% | 64.9% | 64.9% | 75.4% | 64.9% | 68.4% | 68.4% | 75.7% | 66.7% | 70.9% | 64.2% | 82.0% | 66.7% | 82.5% | 64.9% | 65.2% | 64.9% | 100% |  |  |  |  |  |  |
| 30 | 79.5% | 72.0% | 73.3% | 75.8% | 68.1% | 74.5% | 80.7% | 79.5% | 68.9% | 72.0% | 68.9% | 72.7% | 66.0% | 81.1% | 68.1% | 83.6% | 85.5% | 88.7% | 64.3% | 96.0% |  |  |  |  |  |
| 31 | 84.5% | 75.9% | 79.7% | 79.7% | 68.9% | 82.0% | 86.9% | 84.5% | 71.8% | 83.8% | 73.7% | 79.0% | 68.4% | 83.4% | 65.3% | 84.4% | 88.4% | 90.3% | 65.3% | 81.3% | 97.5% |  |  |  |  |
| 32 | 79.7% | 73.9% | 76.1% | 78.3% | 63.0% | 78.3% | 91.3% | 73.9% | 72.8% | 74.6% | 68.4% | 74.7% | 70.0% | 85.5% | 66.7% | 82.6% | 89.9% | 87.0% | 66.7% | 81.7% | 85.9% | 94.5% |  |  |  |
| 33 | 88.0% | 76.7% | 80.0% | 80.0% | 68.0% | 80.0% | 88.0% | 92.0% | 73.0% | 82.0% | 70.8% | 76.9% | 66.7% | 77.0% | 64.0% | 84.0% | 82.6% | 84.0% | 64.0% | 80.0% | 94.7% | 83.5% | 100% |  |  |
| 34 | 92.3% | 83.3% | 88.5% | 88.5% | 69.2% | 80.8% | 80.8% | 88.5% | 75.0% | 90.4% | 78.0% | 82.2% | 66.0% | 77.9% | 65.4% | 76.9% | 80.5% | 80.8% | 65.4% | 74.5% | 87.0% | 77.2% | 91.1% | 100% |  |
| 36 | 91.2% | 80.7% | 85.6% | 86.3% | 67.8% | 80.2% | 85.8% | 87.6% | 73.0% | 85.9% | 75.6% | 79.9% | 65.1% | 78.6% | 64.1% | 80.2% | 80.4% | 84.4% | 64.1% | 78.0% | 90.4% | 81.0% | 94.7% | 95.4% | 95.6% |
|  | 1 | 2 | 3 | 6 | 7 | 8 | 9 | 11 | 12 | 17 | 18 | 20 | 23 | 24 | 25 | 26 | 27 | 28 | 29 | 30 | 31 | 32 | 33 | 34 | 36 |

**Figure S2.** Genetic similarity matrix, comparing the genetic similarity (%) within and among the populations assessed in this study.


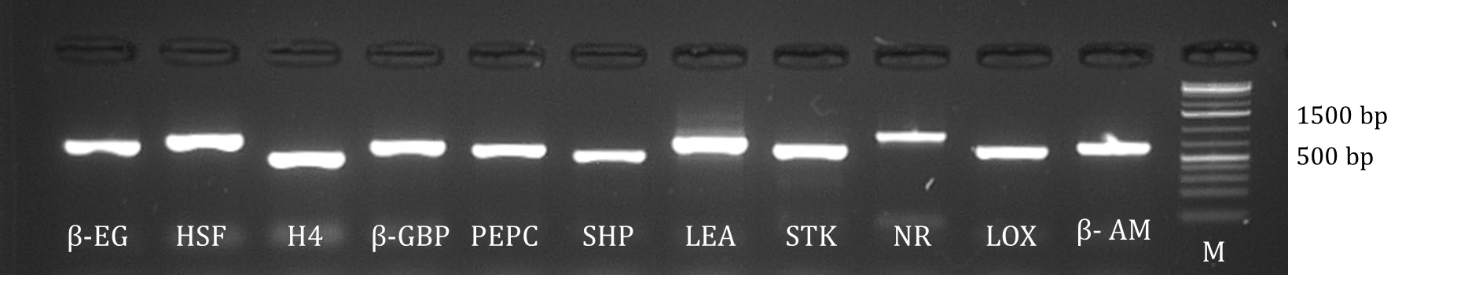


**Figure S3.** Agarose gel electrophoresis analysis of the PCR amplicons used for the analysis in a genomic sample of *Phaseolus vulgaris*. Gene name (codes), marker weight ladders (M), and marker size are reported.
